# Top-View Method as a Robust Alternative for Contact Angle Measurement

**DOI:** 10.1021/acs.langmuir.5c04532

**Published:** 2026-03-04

**Authors:** Emmanuel Agyei, Bimin Zhang Newby

**Affiliations:** Department of Chemical, Biomolecular, and Corrosion Engineering, 1076 The University of Akron, Akron, Ohio 44325-3906, United States

## Abstract

This study validates the top-view method for contact angle measurement, based on the spherical cap assumption, as a practical alternative to conventional side-view techniques. It is particularly useful when side-view imaging is challenging, such as on rough, deformable, irregular, or confined areas where baseline visibility, meridian clarity, or drop symmetry is compromised. To address these challenges, this study details the simultaneous acquisition of top and side-view images of sessile drops along with precise drop volume via drop mass measurements. The contact angles were then deduced from drop volume and contact area based on the spherical cap assumption. Contact angles of water and formamide (1–40 μL) on poly­(methyl methacrylate) (PMMA) and Teflon surfaces, covering both wetting (θ_*s*_ < 90°) and nonwetting (θ_*s*_ > 90°) regimes, were measured. The results demonstrate that the top-view method yields statistically comparable contact angle values to side-view methods for small drops (1–10 μL). The study also introduces a drop projection index (DPI), a dimensionless parameter analogous to the bond number, to quantify gravity-induced effects in top-view imaging. The results showed that for θ_*s*_ < 90°, DPI was ∼0.15 for both formamide and water on PMMA, while for θ_*s*_ > 90°, DPI ranged from ∼0.2 (formamide on Teflon) to ∼0.3 (water on Teflon). Findings in this study provide a foundation for refining the applicability of the top-view method based on the spherical cap assumption and adapting it to a wide range of systems.

## Graphical abstract

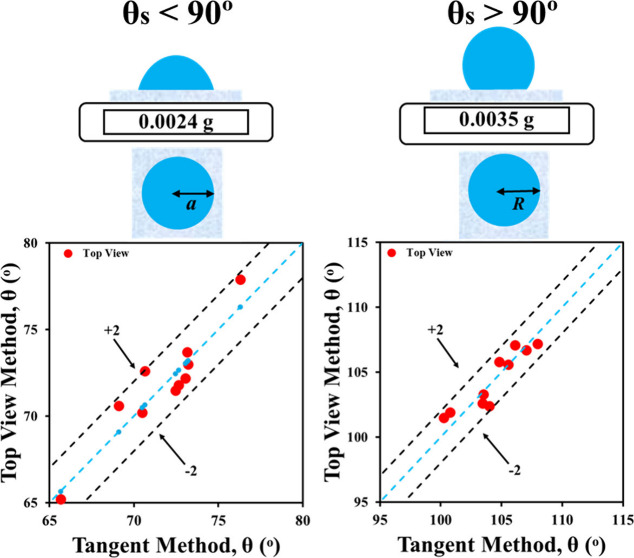

## Introduction

Contact angle, especially the static contact angle (θ_*s*_) measured at the intersection of solid–liquid, solid–vapor, and liquid–vapor interfaces, serves as a key macroscopic indicator of liquid–solid interactions. Its value offers valuable insights into both chemical composition and physical texture (e.g., roughness, topography) of a surface, as well as capillary forces at the micro and nanoscales.^1^ In industries such as semiconductors, pharmaceuticals, coatings, and textiles, θ_*s*_ is used to evaluate surface cleanliness and guide thin film deposition,^2–4^ optimize drug delivery,^5–7^ evaluate adhesion,^8–10^ and develop functional materials.^11–13^ As such, θ_*s*_ remains a critical parameter for characterizing surface wettability and understanding physicochemical properties at interfaces in many industrial applications.^14,15^

Traditionally, the values of θ_*s*_ are obtained using side-view images of sessile drops.^16^ A liquid drop is deposited on a solid surface, and its side view profile is analyzed using geometric methods (e.g., tangent or θ/2 ^17^), or fitting approaches (e.g., circle,^18,19^ ellipse,^20^ polynomial,^21^ B-spline snakes,^22^ or more involved analyses [e.g., Young–Laplace or Axisymmetric Drop Shape Analysis (ADSA),^23,24^ Low-Bond Axisymmetric Drop Shape Analysis (LB-ADSA)^25^]. While these techniques vary in complexity, they all rely on clear side-view images with a visible, well-defined baseline. However, such conditions are not always attainable, especially on rough, deformable, porous surfaces or in confined spaces, leading to blurred profiles and obscured baselines that compromise the accuracy of the side-view techniques. As a result, there is an increasing interest in alternative approaches, such as top-view imaging for contact angle measurement.

The earliest attempt at a top-view method for measuring θ_*s*_ was introduced by Skinner et al.,^23^ who developed ADSA-CD (CD: Contact Diameter), which numerically solves the Young–Laplace equation using the measured top-view CD, fluid surface tension, and drop volume. They obtained θ_*s*_ (<90°) of 40 and 7 μL of ethylene glycol and undecane drops, respectively, on siliconized glass slides. The ADSA-CD method yielded results within ±0.5° of those side-view methods (ADSA-P, P: profile, and tangent). Moy et al.^24^ extended the top-view approach to drops with θ_*s*_ > 90° by using the drop’s maximum horizontal diameter (MD), since CD cannot be observed directly from the top-view image. The ADSA-MD method was tested on hydrophobic polymer films and on the intestine of rabbits using ∼89 μL water drops, yielding results comparable to ADSA-P.

Despite the accuracy of top-view ADSA methods in obtaining contact angles for large drops, later studies reported inconsistencies when the first and second generations of ADSA techniques are applied to spherical or near-spherical drops,^26^ which are common in contact angle measurements. These reported inconsistencies raise concerns about the applicability of ADSA-CD and ADSA-MD beyond the gravity-distorted drops. Also, these ADSA top-view methods are not available in open-source software like ImageJ, limiting accessibility. Therefore, an alternative technique is explored in this study.

The alternative explored in this study is a top-view method based on the spherical cap assumption. The spherical cap assumption has traditionally been applied to side-view images for obtaining contact angles.^27^ It assumes the drop forms a segment of a perfect sphere. The model requires any two of three measurable parameters: base radius (*a*), drop height (*h*), and volume (*V*), to compute the contact angle, and is therefore often referred to as a two-parameter spherical cap model. While previously used for evaporating drops with a side-view profile,^28–31^ this study adapts the model for top-view imaging (using eqs 1 & 2, respectively, for θ_*s*_ < 90° and θ_*s*_ > 90°, with *R* being the drop radius or maximum horizontal radius). Figure 1a and b depict a typical side and corresponding top-view of a sessile drop based on the spherical cap model, for θ_*s*_ < 90° and θ_*s*_ > 90°, respectively. From a side view, either radius (*a* or *R*) or drop height (*h*) can be measured directly, so either of them could be used along with drop volume (*V*) to obtain the contact angle. However, the only parameter that can be measured directly from a top-view image of a drop is the radius (*a* for θ_*s*_ < 90° or *R* for θ_*s*_ > 90°). As a result, equations relating drop volume to *a* (eq 1) and relating to *R* (eq 2) will be applied, respectively, to obtain θ_*s*_ for θ_*s*_ < 90° or for θ_*s*_ > 90° in this study for the top-view images. These two equations can be further modified to use drop mass, which could increase the accuracy of determining drop volume, instead of the direct pipetting of drop volume.

1 $$V=\frac{\pi a^{3}\left(2-3\cos\theta_{s}+\cos^{3}\theta_{s}\right)}{3\sin^{3}\theta_{s}}$$

2 $$V=\frac{4}{3}\pi R^{3}-\frac{\pi R^{3}}{3}\left(2+3\cos\theta_{s}-\cos^{3}\theta_{s}\right)=\frac{\pi R^{3}}{3}\left(2-3\cos\theta_{s}+\cos^{3}\theta_{s}\right)$$

**Figure 1:**
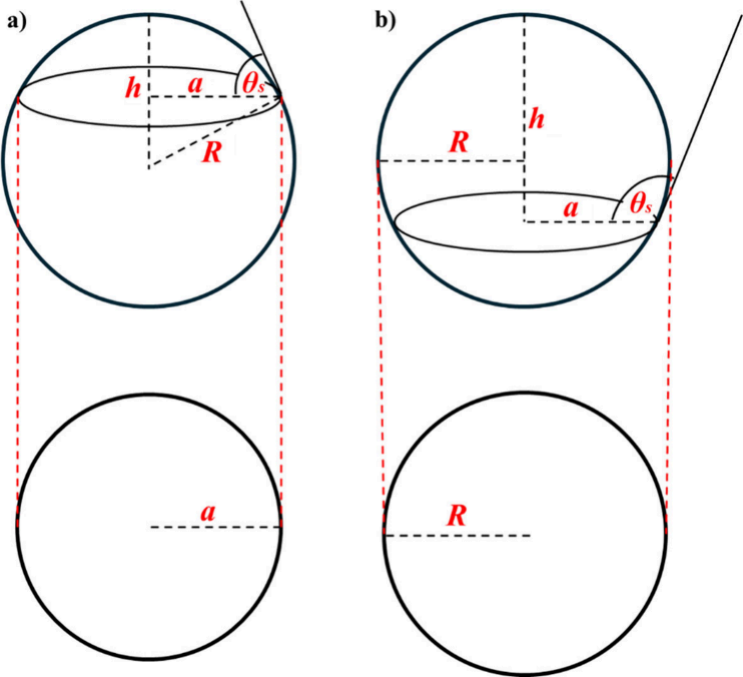
Based on the spherical cap assumption, side and top view geometries of a sessile drop with (a) the static contact angle, θ_s_ *<* 90° and (b) θ_s_ > 90°, are respectively illustrated.

Several studies have utilized the top-view method based on the spherical cap assumption for contact angle (θ_*s*_) measurements. Dutra et al.^32^ determined water contact angle on hydrophilic glass using ∼2 μL drops, and the results had ±6° to ±8° error margins. Zhang and Chao^33^ used the spherical cap model but did not comprehensively validate its applicability for top-view contact angle estimation. Janeczko et al.^34^ developed an image recognition model based on spherical cap for enhanced contact angle estimation and surface mapping, but they tested only a single drop volume (∼4 μL) and fluid (water). These studies focused on either below or above 90° with narrow volume ranges, and do not address key questions about the broader applicability of the method. A summary of relevant studies on the implementation of top-view methods for obtaining contact angles is presented in Table 1. Specifically, it remains unclear whether the top-view measurements can reliably match side-view results, under what conditions they are accurate, and what limitations exist. This study, therefore, evaluates whether the top-view method, based on the spherical cap assumption, can serve as a practical and dependable alternative in situations where side-view imaging is technically challenging to obtain.

**Table 1:** Relevant Literature to This Study

Study reference	Measured Quantity^a^	Key Assumptions	Advantages	Limitations in Relation to this Study
Skinner et al.^23^	2*a*, V, and g	Axisymmetric drop, circular contact line.	geometry-independent, physically rigorous, high accuracy (∼ ± 0.5°), ideal for low θ_*s*_(<30°).	1. Neither assessing the top-view method nor the influence of gravity.
				2. Only valid for θ_*s*_ < 90°
Moy et al.^24^	R, V, and g	Axisymmetric drop	geometry-independent; explicitly accounts for gravity.	1. Used large drops (∼90 μL) greatly affected by gravity.
				2. Only valid for θ_*s*_ > 90°.
McHale^35^	Top-view drop shape	Ellipsoidal drop profile	Explicit top-view imaging reveals drop asymmetry not detectable from the side view.	1. not a standalone top-view θ_*s*_ measurement method.
				2. θ_*s*_ still from side-view profiles.
Zhang and Chao^33^	V and 2*a*.	spherical-cap for small static drops.	Independent of side-view profile, applicable to nontransparent substrates; capable of measuring dynamic θ.	1. primarily for the dynamic *q* of evaporating drops.
Rodriguez-Valverde et al.^36^	R	Axisymmetric drop.	Applicable to nonideal surfaces; yielding averaged θ_*s*_ for irregular contact lines; applicable to wood, and stone	1. not applying the top-view method.
Dutra et al.^32^	2*a*	Spherical-cap; accurate *V*; negligible gravitational deformation.	Simple and cost-effective; top-view imaging with known *V*; no baseline placement is needed;	1. Strictly for the spherical-cap regime w. θ_*s*_ < 90°.
				2. Only a single small *V* (2 μL) was attempted.
Schuster et al.^37^	incircle/excircle ratio	*V* is estimated from a spherical cap.	highlights top-view inspection for symmetry; introduces an asymmetry coefficient for reliability; examines key variables affecting θ_*s*_.	1. θ_*s*_ is obtained from side-view rather than top-view.
Janeckzo et al.^34^	2*a*	spherical-cap valid for small drops.	nondestructive top-view θ_*s*_ mapping on nonideal surfaces; automated;	1. dependent on reliable image processing and lighting.
				2. θ_*s*_ vs *V* not quantified.

a Note: “Measured quantity” denotes variables measured in the study to either estimate or validate the measured contact angles.

These gaps in literature highlight the need to evaluate the top-view method based on spherical cap assumptions more thoroughly. In this study, we designed an experimental framework that enables simultaneous acquisition of side-view and top-view images, along with precise drop mass. We tested two wetting and two nonwetting surfaces using four drop volume ranges: 1–5 μL, 5–10 μL, 10–20 μL, and 20–40 μL. The results obtained allow for a comprehensive comparison between top-view and side-view contact angle measurements, helping to identify the conditions under which the top-view method is accurate and reliable. Furthermore, we introduced dimensionless shape descriptors to quantify gravitational effects and define the operational limits of the top-view method under the spherical cap assumption.

## Experimental Section

### Materials and Equipment

Poly­(methyl methacrylate) [PMMA; avg Mw = 120 K] and Teflon tape were purchased from Sigma-Aldrich and CFPC, respectively. Deionized (DI) water (conductivity value of <2 μS/cm) prepared in-house, and formamide [Super pure; Mw = 45.04], purchased from Research Products International (RPI), were chosen as probe liquids for this study. These liquids were selected because of their availability and well-defined chemical and physical properties. Water and formamide also provide the range of contact angles (greater than or less than 90° on the selected surfaces) needed for this study. The density and surface tension, along with its Lifshitz-van der Waals (γ^*LW*^ and Lewis acid–base (γ^*AB*^) components of water and formamide are summarized in Table 2.

**Table 2:** Relevant Properties of Probe Liquids Used in This Study at 23 °C^38,39^

Liquid	Molecular diameter (Å)	ρ (kg/m^3^)	γ (N/m)	γ^*LW*^ (N/m)	γ^*AB*^ (N/m)
Formamide	∼4.7	1134	58.36	31.72	26.64
Water	∼2.7	998	72.80	21.80	51.00

Equipment and other materials used in this study include micro glass slides (1 mm thick), which was obtained from VWR International LLC, P-6000 spin coater (Specialty coating systems Inc.), Intel Core i7 HP laptop, Digital USB Teslong microscopes (10x–200× magnification), analytical balance (with an accuracy of 0.1 mg, OHAUS explorer series), Petri dish, Eppendorf Digital Pipette 4710 distributed by Brinkman Instruments, Inc., VWR pipet and sterile aerosol pipet tips with low adhesion for 20 μL pipettors.

### Sample Preparation

Glass slides (25 × 25 × 1 mm) spin-coated with ∼120 nm of PMMA or covered with Teflon thread tape were used as substrates. These polymers were chosen for their availability and to represent a range of contact angles. Prior to coating, glass slides were cleaned using a freshly prepared piranha solution (30% H_2_O_2_/concentrated H_2_SO_4_, 30/70 v/v), rinsed thoroughly with deionized (DI) water, and stored in a DI water bath. The polymer solution was prepared by dissolving approximately 2 wt.% of PMMA in toluene. ∼200 μL of this solution was deposited on each slide before spin-coating at 2000 rpm for 60 s. Three PMMA-coated samples were prepared and dried in a fume hood, then stored in clean Petri dishes. For Teflon samples, three clean glass slides were covered with a thin layer of Teflon tape and stored similarly.

### Experimental Setup

The spherical cap model is highly sensitive to drop volume (see Figure S1), making precise volume measurement essential for accurate contact angle determination. To avoid inconsistencies from pipet-based dispensing, this study used a mass-based approach, measuring drop mass with a 0.1 mg-precision analytical balance.

The experimental setup (Figure 2) featured a dual-camera system for simultaneous top and side-view imaging. The sample was placed on the balance, enclosed by Plexiglas and plastic sheets to minimize air currents. Microscopes (with cameras) with built-in illumination were mounted on adjustable stands, with cutouts in the enclosure for optimal viewing. A Petri dish was placed on the sensitive scale of the balance to provide an additional platform (height) for positioning the sample to align with both cameras. The setup rested on a vibration-dampened lab bench, and experiments were conducted at ∼23 °C and ∼50% relative humidity.

**Figure 2:**
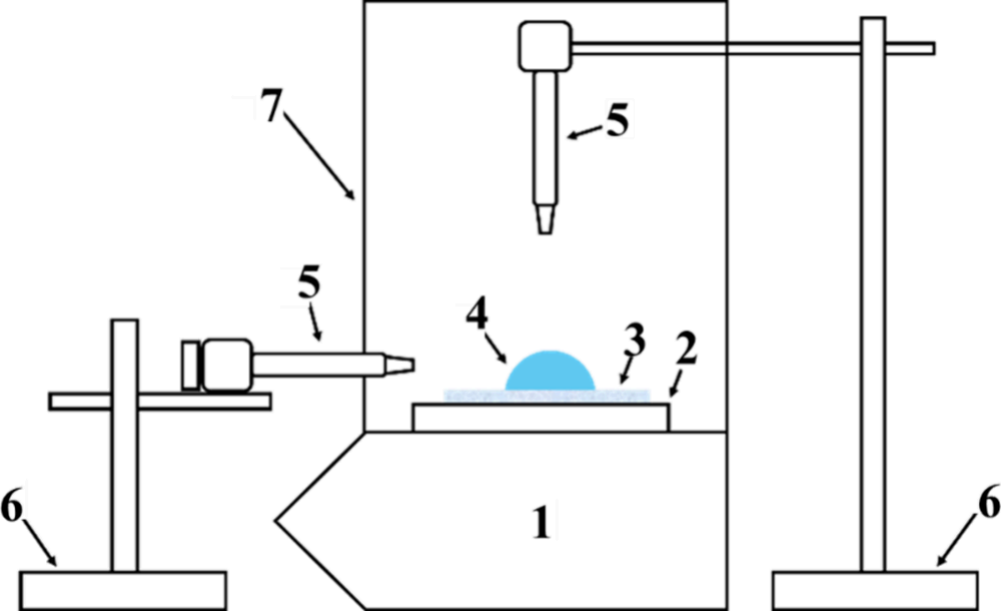
Schematic of experimental set up for simultaneously measuring the mass of the drop place on a solid surface and capturing of side and top views of a liquid drop: (1) OHAUS Explorer analytical balance with an accuracy of 0.1 mg, (2) sample stage, (3) sample, (4) liquid drop, (5) microscopes, (6) microscope stand assemblies, and (7) modified glass doors for an enclosure.

### Image Acquisition and Analysis

The procedure began with setting up the image-capturing system. Two microscope cameras and an Ohaus Explorer analytical balance were connected to the laptop, enabling synchronized image and mass data collection. The balance was leveled, and the total mass of the sample stage with the sample on it was zeroed to ensure only the mass of the dispensed drop mass was recorded during the experiment. A digital Eppendorf pipet (2–20 μL range) with low-adhesion tips was used to deliver drops onto the sample surface.

Drops were dispensed in four volume groups: 1–5 μL, 5–10 μL, 10–20 μL, and 20–40 μL (with water on Teflon extended to 60 μL to ensure the drop radius is close to or greater than the capillary length of the liquid). These drop volume ranges were chosen to probe the transition from surface-tension-dominated to gravity-influenced drop regimes systematically. Volumes below ∼5 μL are widely accepted in literature as satisfying the spherical-cap assumption, where gravitational deformation is negligible. Intermediate ranges (5–10 μL and 10–20 μL) were selected to progressively capture the onset of gravity-induced deviations from ideal spherical geometry, which has been a common concern in contact-angle measurements. The largest volume group (20–40 μL, extended to 60 μL for water on Teflon) was included to ensure drop radii approach or exceed the capillary length of the liquid, a regime historically used in classical studies to explicitly examine gravitational effects. This stepwise volume grouping, therefore, enables a controlled, quantitative assessment of nongravity and gravity-dominated effects on top-view contact-angle determination based on the spherical cap assumption. Each group included 10 drops per fluid–sample combination, totaling 40 drops per system. The top and side-view images of the drop, along with its mass, were captured simultaneously. 5–10 images were taken from both views, and mass was recorded for 10–15 s postdeposition. These volume groups allowed analysis across both surface tension- and gravity-dominated regimes.

### Contact Angle Data Acquisition

Contact angles for all four liquid-sample combinations were obtained using the proposed top-view method and selected conventional side-view techniques. All analyses were performed in ImageJ, an open-source software developed by the National Institute of Health and the Laboratory for Optical and Computational Instrumentation.^40^ Side-view images were processed using ImageJ plugins for the tangent method, circle fit, ellipse fit, and LB-ADSA, with multiple measurements taken to minimize operator error. For both the top-view and side-view analyses, the drop boundary was fitted using the circular selection tool in ImageJ, with the fitting circle determined manually by aligning it to the visually identified drop perimeter. Because the calculated contact angle is highly sensitive to small variations in the fitted radius, particular care was taken during circle placement, including repeated visual verification of boundary alignment to minimize operator-dependent uncertainty. While manual circle fitting introduces user dependence, this limitation is explicitly acknowledged in the present work and is considered in the interpretation of the results.

For top-view analysis, the drop perimeter, from the image, was carefully traced using the oval selection tool in ImageJ to ensure accurate delineation of the drop outline. The enclosed area was then measured and assumed to correspond to a circle, from which the equivalent radius, *a* or *R* $\left(=\sqrt{A/\pi }\right),$ was calculated This radius was subsequently used in either eq 1 or eq 2, along with known drop volume (from the measured mass and liquid density), to obtain the contact angle. eq 1 was used for contact angles less than 90° (θ_s_ < 90°), and eq 2 for θ_s_ > 90°. The tangent method, with a typical ± 2° error margin, served as the benchmark for comparisons. Since θ_*s*_ appears inside nonlinear trigonometric terms in eqs 1 and 2, it is solved expeditiously with the aid of Excel Solver using the measured volume and radius.

### Statistical Analysis

Independent sample *t* tests were used to assess the accuracy, consistency, and comparative performance of the top-view contact angle method compared to those side-view techniques (LB-ADSA, circle fit, ellipse fit, and the tangent method). Pairwise comparisons were conducted to compare each method against the tangent technique, which served as the benchmark due to its simplicity and widespread use. All tests were conducted at a significant level of α = 0.05. These *t* tests provided a robust evaluation of the top-view method’s reliability and helped identify techniques that significantly deviated from the top-view or tangent method.

## Results and Discussion

### Representative Images

Figure 3 presents representative side-view (a–d) and corresponding top-view (e, f) images of water drops on PMMA. The side-view profiles reflect moderate wetting behavior of water on PMMA, consistent with reported values of 60–75°.^41,42^ As drop volume increases, the shape transitions from a spherical cap to a more flattened profile, indicating increased influence of gravity and contact-line pinning.^35,43^ For small drops, 1–5 μL (3 μL shown in Figure 3a), the side-view profiles closely match circular segments, indicating surface tension domination and minimal gravitational distortion, and they exhibit spherical cap geometry.^35,44^ Corresponding top-view images (Figure 3e) show a circular and symmetric shape with sharp edges, supporting the notion that the drop is axisymmetric and geometrically undistorted.

**Figure 3:**
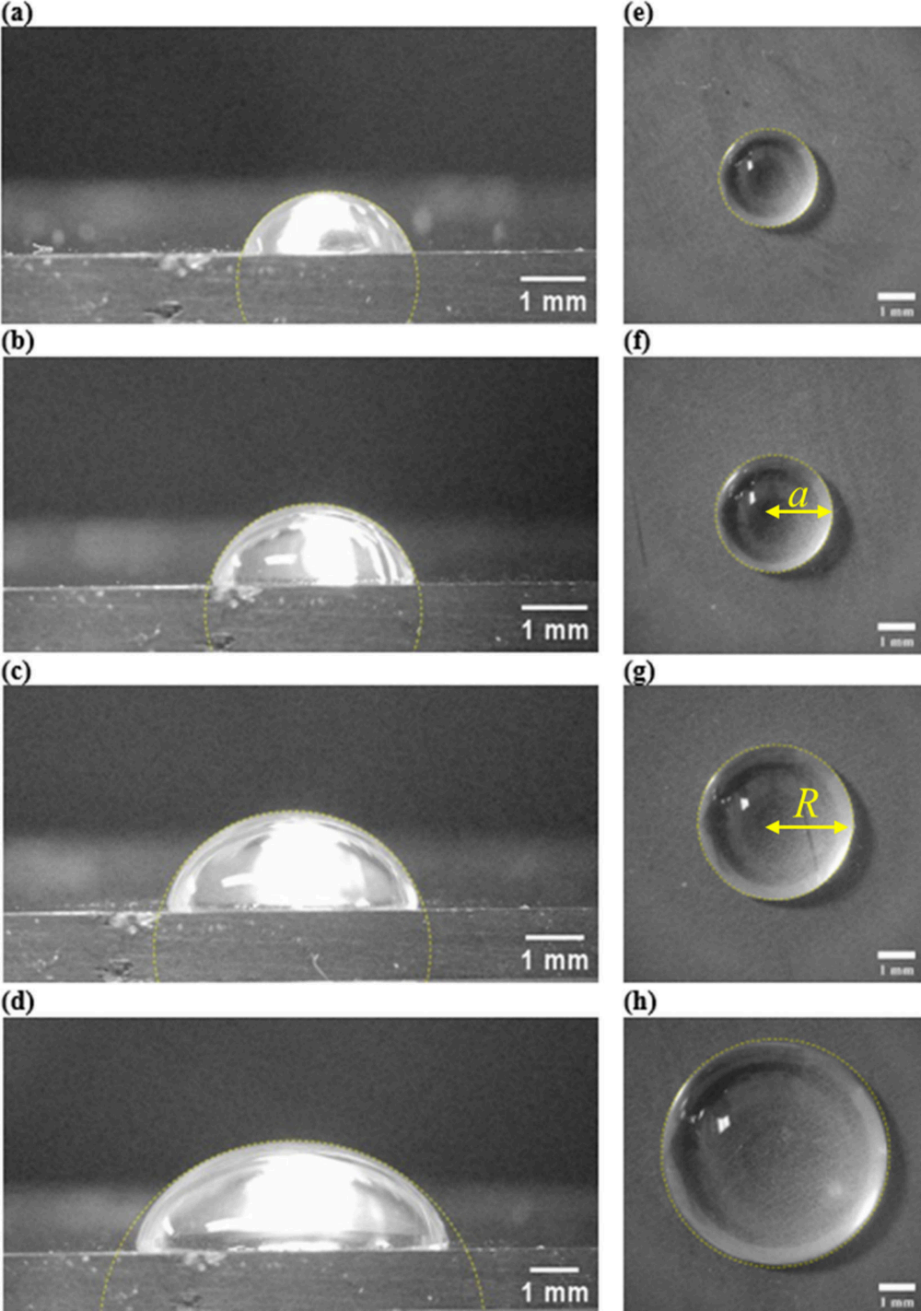
Typical side-view and corresponding top-view images of 3 μL (a, e), 5.6 μL (b, f), 14.8 μL (c, g), and 33.9 μL (d, h) water drops on a PMMA surface. A partially fitted circle for the side-view image, a yellow dashed line, with radius corresponding to the radius of curvature of the drop apex, is drawn for each drop to help visualize the drop distortion from sphericity.

For water drops between 5–20 μL (Figures 3b,c and 3f,g), side-view profiles begin to deviate from the fitted circle, particularly near the base, while the apex appears to retain its curvature. This observation suggests increasing gravitational influence and the onset of contact line pinning. Despite these changes, the top-view images remained mostly circular and symmetrical. At larger volumes (20–40 μL; Figures 3d and 3h), the apex becomes visibly flattened, and the drop profile deviates from the circular fit across both upper and lower portions of the side-view profile. However, the top-view shape remains broadly circular. Similar volume-dependent profile changes were observed across all fluid-surface combinations (see Figures S2, S3, and S4).

### Gravitational Flattening and Contact Line Pinning on Side-View and Top-View Profiles

Given the observed changes in drop profiles with increasing volume, the effects of gravity and contact line pinning on both side-view and top-view drop profiles, and thus contact angles, were evaluated. While their influence on sessile drop side-view profiles, and consequently contact angles, is well documented,^35,43^ their impact on top-view profiles and contact angles is much less understood. Therefore, it is important to assess how gravity and contact line pinning affect top-view measurements.

When a drop sufficiently large enough to experience gravitational flattening is deposited on a surface, three scenarios may arise (Figure 4a). In this case, the drop deviates from an ideal spherical-cap geometry, and the three-phase contact line advances. The resulting apparent static contact angle (θ_*s*_) is governed by the interplay between drop spreading influenced by gravity, liquid–solid interactions, as well as resistance to contact line movement due to pinning.

**Figure 4:**
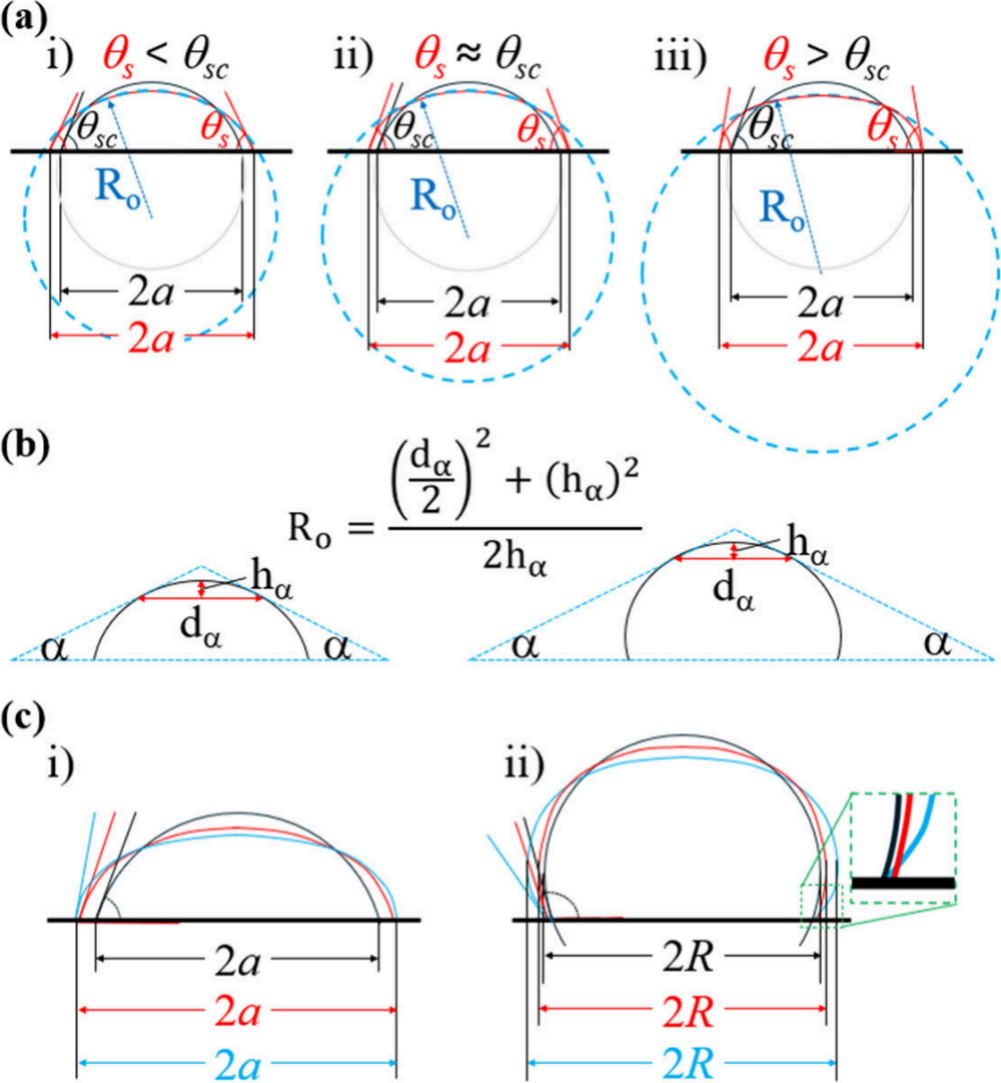
(a) and (c) demonstrate possible ways drop properties could be affected by gravity and contact line pinning. (b) The technique used for obtaining the radius of curvature at the drop apex, *R*_o_, for all drops used in this study. This technique is modified from the Dorsey method.^45,46^

The static contact angle reported in this study is an apparent, experimentally measured quantity that may be influenced by contact line pinning. It is different from the equilibrium contact angle, which is a thermodynamic quantity defined by Young’s equation in the absence of pinning and hysteresis. Even with spreading, the intrinsic wettability of the surface dictates the equilibrium tendency of the contact angle and, in the absence of pinning, θ_*s*_ should follow Young’s equation, making scenario 1 (θ_*s*_ < θ_*sc*_), where θ_*sc*_ is the contact angle of a spherical capped drop, unlikely.

Scenario 2 corresponds to conditions under which contact-line pinning is negligible, allowing the contact line to advance freely and yielding θ_*s*_ ≈ θ_*sc*_ as observed by Extrand and Moon.^43^ In practice, however, some degree of three-phase contact line pinning is typically present. Pinning restricts further motion of the contact line while gravitational flattening continues, leading to an increase in the apparent static contact angle or scenario 3 (θ_*s*_ > θ_*sc*_).

Gravitational flattening and contact line pinning become evident in side-view images for drops with volumes larger than 10 μL. To assess these effects using top-view images, the radius of curvature at the apex (*R*_*o*_) and the contact radius (*a*, for θ_*s*_ < 90°) or drop radius (*R*, for θ_*s*_ > 90°) were collected. *R*_*o*_ was determined using a slightly modified Dorsey method (Figure 4b). Specifically, an isosceles triangle is constructed using a small angle (α ≤ 30°), with its two sides forming tangents at points near the apex, from which *R*_*o*_ was determined. *a* and *R* were measured experimentally from the top-view images, as described in the Contact Angle Data Acquisition section.

For comparison, the expected radii for an ideal spherical-cap, *a*_e_ and *R*_*e*_, were estimated with the given drop volume and the average contact angle values obtained from the side-view images for each probe liquid-polymer pair using eq 1 and eq 2, respectively. For θ_*s*_ < 90°, corresponding *R*_*e*_ from *a*_e_ was also estimated using *R*_*e*_ = *a*_e_/sin θ_*s*_. The contact angles were measured using small drop volume (1 – 5 mL), for which the gravitational effects are negligible. The experimentally measured values (*a* or R) from the ideal predictions (*a*_e_ or *R*_*e*_) are presented in Figure 5. We expect that the magnitude of these deviations correlates to the extent of gravitational influence and contact line pinning present in the drop.

**Figure 5:**
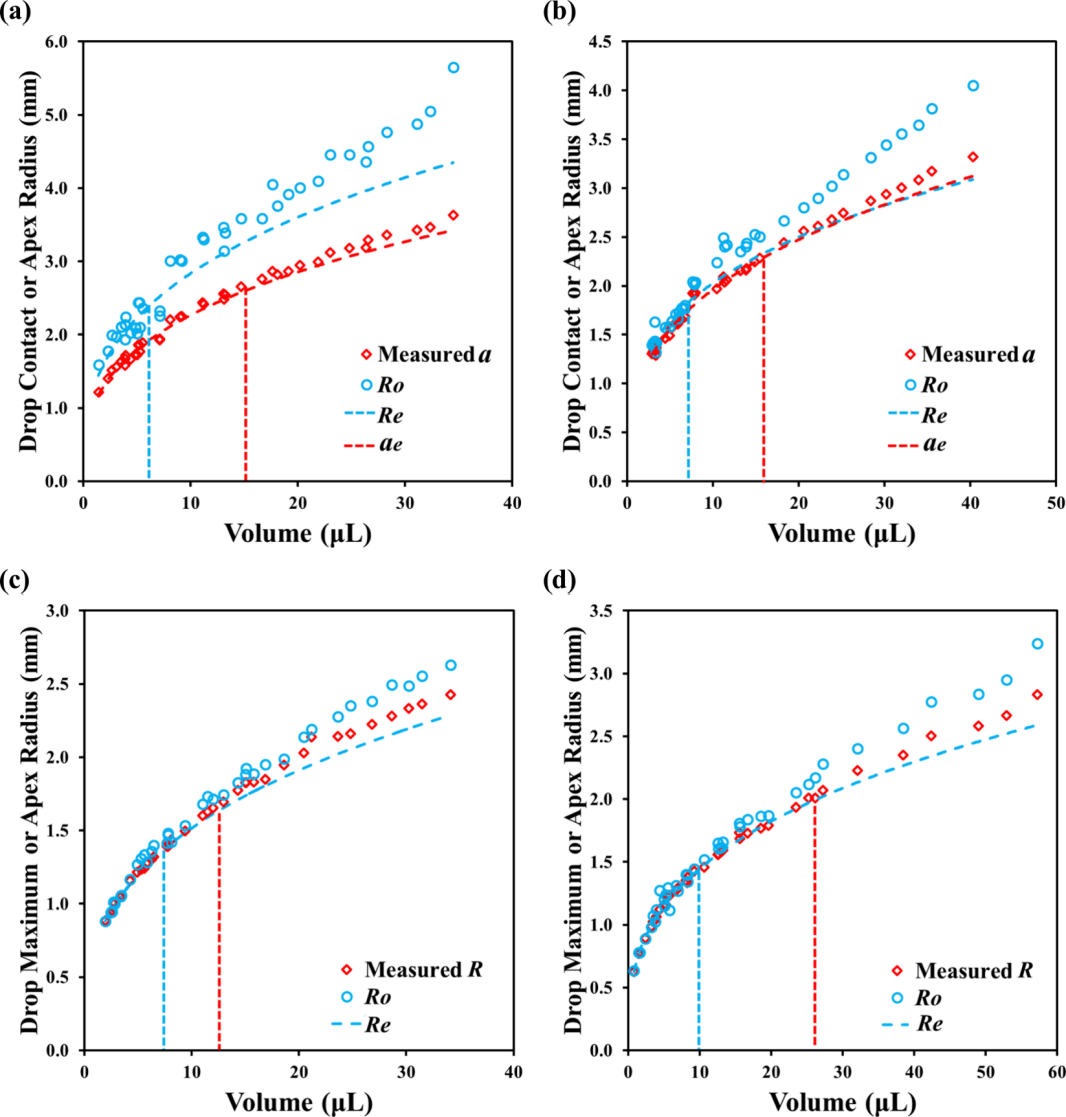
Comparison of the measured top-view radius (*a* for θ_s_ *<* 90°, or *R* for θ_s_ > 90°) and side-view *R*_o_ with the expected spherical cap radius, *R*_e_, for the four fluid–sample systems: (a) formamide on PMMA, (b) water on PMMA, (c) formamide on Teflon, and (d) water on Teflon. For drops with θ_s_ *<* 90°, *a* values are also compared to their corresponding spherical cap estimations. The vertical dotted lines serve as visual guides to show regions in which the measured values, to the right of the lines, exceed the expected values.

Across all systems, measured and predicted values align closely at small drop volumes but begin to deviate as volume increases. By examining the data when the measured values consistently exceeded the predicted values over a continuous range of drop volumes, the difference between the measured and the predicted values was found to be at least 2% and continued to increase as the drop volume increased. We therefore adopted a 2% deviation as a quantitative criterion for defining the threshold drop volume. Under this criterion, for formamide on PMMA (Figure 5a), both *a* and *R*_*o*_ align well with predicted values at small volumes, i.e., <8 μL for *R_o_* and <15 mL for *a*. Beyond these volume ranges, noticeable deviations emerge. The comparatively better agreement, or smaller deviation, observed for *a* suggests either reduced lateral gravitational distortion or stabilization of the contact line through pinning or favorable surface energy conditions that resist gravity-driven spreading. These observations indicate that gravity primarily induces vertical flattening of the drop rather than significant lateral expansion.

Figure 5b (water on PMMA) shows a similar trend but with a greater deviation than formamide at higher volumes. *R*_*o*_ again deviates more, as compared to *a*, from the predicted values. The observation suggests greater gravitational distortion, for larger drops, in both vertical and lateral dimensions for this system, likely due to lower wettability, i.e., higher contact angle, smaller contact area, and increased drop height. As wettability decreases, the resulting hydrostatic pressure due to increased drop height may flatten the drop.^44^

For drops with θ_*s*_ > 90°, i.e., formamide and water on Teflon (Figure 5c, d), measured radii also closely matched predictions at lower volumes, with deviation for large drops. The threshold volume when the gravitational flattening appeared increases with the increase of θ_*s*_. As before, more deviation occurred for *R*_*o*_, reaffirming that gravitational flattening primarily affects the vertical profile.

### Top-View Method Performance of Our Model Systems

Figure 4c illustrates how gravitational flattening and contact line pinning affect both top- and side-view drop profiles, and consequently, the measured contact angles, as drop volume increases. Three scenarios are depicted: (1) drops maintain as spherical caps (black), (2) drops flattened without pinning, resulting in increased *a* or *R* (red), and (3) drops flattened with contact-line pinning (blue) leading to minimal or no increase in *a* but a more pronounced increase in *R*. From the side-view, drops without contact line pinning would retain contact angles as those of the spherical cap drops. In contrast, pinning resists drop spreading and would lead to an increase in apparent contact angle until it reaches its advancing contact angle (see Table S1).

In the top view, contact angle progression is inferred from changes in *a* (contact radius) and *R* (maximum horizontal radius), both influenced by gravitational flattening and pinning. As shown in Figure S1, contact angles obtained from top-view images are sensitive to these radii. Figure 4c illustrates how gravity and contact line pinning affect top-view radii. For θ_*s*_ < 90°, gravitational flattening increases *a* if no pinning, thus decreasing the obtained contact angle. With pinning, *a* could still increase slightly, also leading to a decrease in contact angle. For θ_*s*_ > 90°, gravity alone causes minimal increase in *R*, but when combined with pinning, *R* can increase noticeably, leading to underestimation of the contact angle in top-view analysis. Thus, for large drops, gravitational flattening with contact line pinning leads top-view measurement to underestimate the contact angle.

A summary of average contact angles for side view (tangent method) and top-view approach, with increasing drop volume, is presented in Table 3. For formamide on both surfaces, the side-view resulted in relatively consistent contact angles across different drop volumes, whereas the top-view had a noticeable decrease in contact angle with increasing volume, particularly on Teflon.

**Table 3:** Average Contact Angles of All Fluid Substrate Combinations Obtained Using Side-View (Tangent) and Top-View Approaches

		contact angle, θ_s_ (°)			
Fluid-substrate	method	1–5 μL	5–10 μL	10–20 μL	20–40 μL
Formamide on PMMA	side	53.5 ± 1.0	54.8 ± 1.4	52.6 ± 0.8	52.5 ± 1.8
	top	53.2 ± 1.0	53.7 ± 1.4	50.4 ± 0.4	49.3 ± 1.4
Water on PMMA	side	71.4 ± 1.1	70.7 ± 1.5	74.1 ± 0.8	75.9 ± 1.0
	top	71.9 ± 1.2	70.1 ± 1.5	71.3 ± 0.3	66.7 ± 1.7
Formamide on Teflon	side	104.3 ± 0.8	104.3 ± 0.3	104.8 ± 0.3	105 ± 1.5
	top	104.4 ± 0.7	103.6 ± 0.3	99.2 ± 0.5	95.4 ± 1.5
Water on Teflon	side	113.9 ± 0.8	114.4 ± 0.9	121.4 ± 1.2	120.9 ± 2.4
	top	113.3 ± 0.8	111.9 ± 0.8	113.5 ± 0.8	106.3 ± 4.1

For water, side-view contact angles increase with volume, while top-view angles decrease at higher volumes. As stated above, the reduction in contact angles at larger volumes is likely due to an increased *a* or *R*. When comparing water to formamide, the increase in water’s contact angle with volume is likely attributed to its higher contact angle hysteresis on these surfaces (see Table S1). Liquids like water, which have a smaller molecular size and a higher polar surface tension component (Table 1), tend to exhibit greater contact angle hysteresis.^47,48^

When comparing the two methods, top-view contact angles closely matched side-view results for all fluid–sample combinations at small volumes (1–5 μL), with most values within ±2° of the tangent method and agreement in at least 9 out of 10 drops (Figure 6). Statistical analysis (Figure S5) showed no significant differences (*P* > 0.80), except for formamide on Teflon, where the ellipse fit method yielded consistently higher angles, likely due to overestimation by the ImageJ plugin at high contact angles. These results support the validity of the spherical cap assumption at small drop volumes, where surface tension dominates, and gravitational effects are minimal.

**Figure 6:**
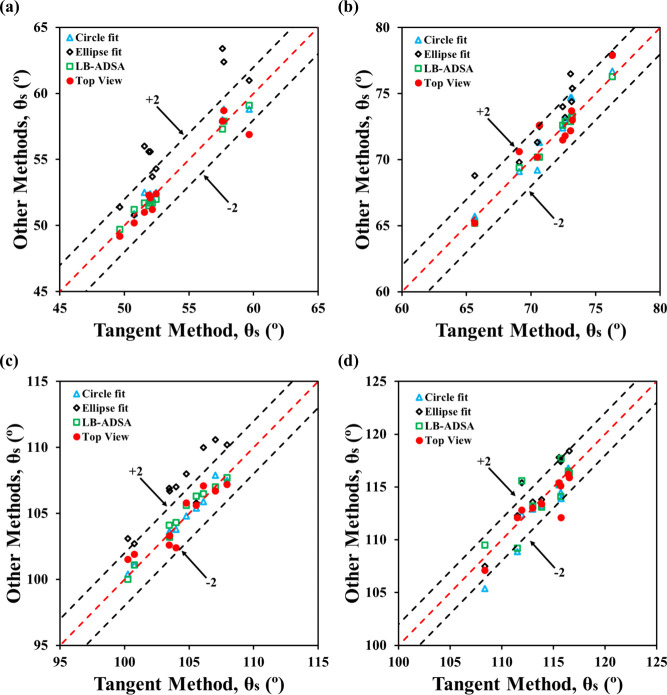
Performance of the top-view method against conventional side-view methods for 1–5 μL drops: (a) formamide on PMMA, (b) water on PMMA, (c) formamide on Teflon, and (d) water on Teflon.

In the 5–10 μL range, the top-view method remained reasonably accurate but began to show deviations, especially for θ_*s*_ > 90°. For most systems, 9 out of 10 top-view measurements stayed within ±2° of the tangent method (Figure 7), except for water on Teflon, where only 5 out of 10 met this threshold. Statistically, top-view and circle fit methods showed no significant differences (*p* > 0.55), reflecting their shared reliance on spherical geometry. However, comparisons with the ellipse fit method revealed significant discrepancies for high-contact-angle cases (θ_*s*_ > 90°), with p-values <0.05. These trends suggest that while the top-view method remains valid in this volume range, its accuracy becomes increasingly sensitive to contact angle and gravitational effects. Notably, contact angles in the 5–10 μL range were not significantly different from those in the 1–5 μL range, indicating minimal pinning and limited gravitational distortion.

**Figure 7:**
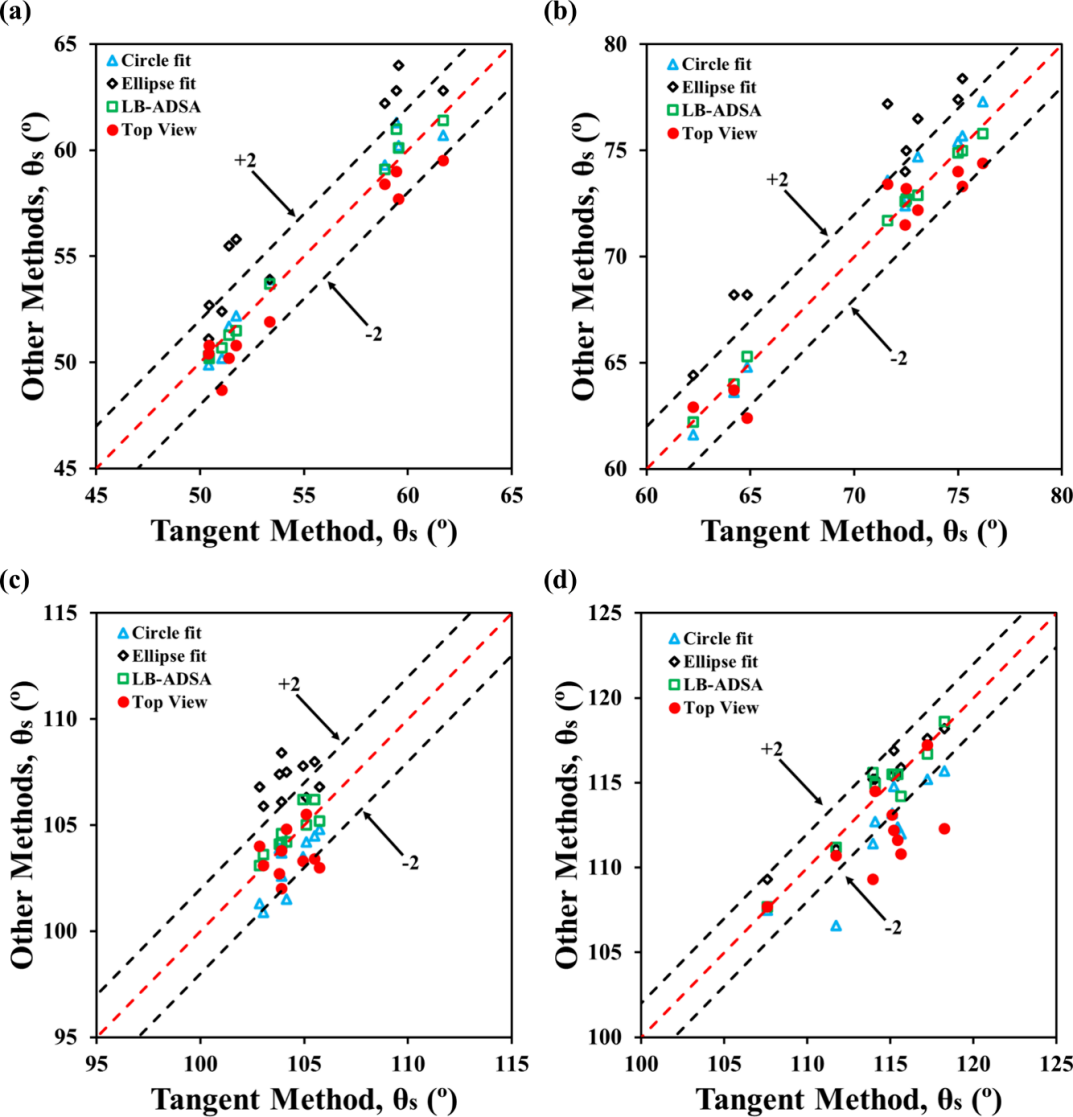
Performance of the top-view method against conventional side-view methods for 5–10 μL drops: (a) formamide on PMMA, (b) water on PMMA, (c) formamide on Teflon, and (d) water on Teflon.

In the 10–20 μL range, gravitational distortion became more evident, especially in side-view images, leading to reduced accuracy of the top-view method across all fluid–sample combinations. Only half of the top-view measurements for θ_*s*_ < 90° fell within ± 2° of the tangent method (Figure 8a, b), and significant differences were found between top-view and most side-view techniques (*p* < 0.05). For θ_*s*_ > 90°, all top-view estimates exceeded the error margin. These results highlight the limitations of top-view method at larger volumes due to breakdown of the spherical cap assumption and inability to capture vertical flattening. While side-view contact angles for formamide remained consistent across volume groups, top-view estimates decreased due to increased base radius. In contrast, water contact angles were close to their advancing angles at higher volumes, indicating combined effects of gravity and pinning. Interestingly, top-view contact angles for water, presumably underestimated due to greater *a* or *R* measured, did not differ significantly from smaller volume groups.

**Figure 8:**
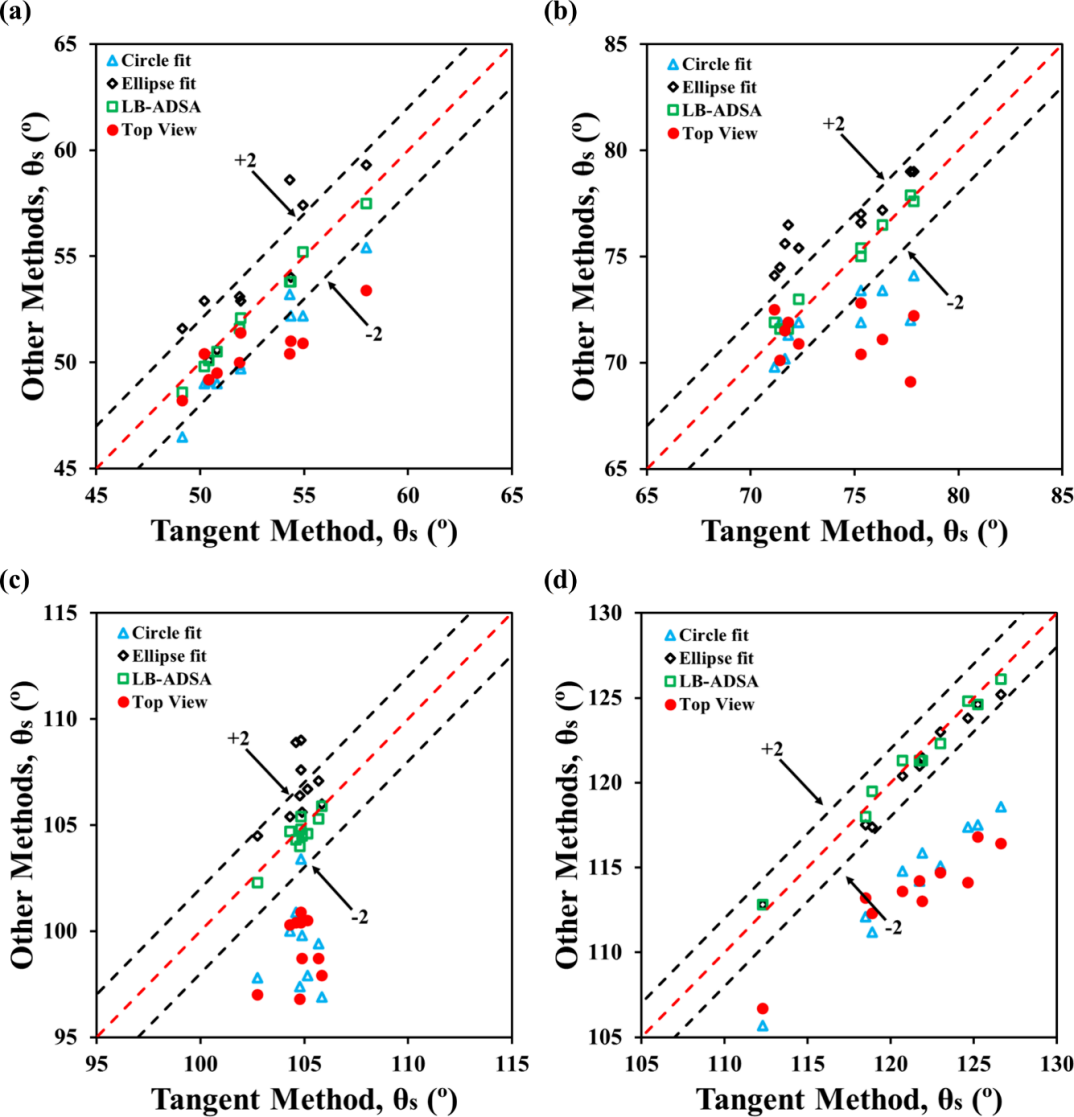
Performance of the top-view method against conventional side-view methods for 10–20 μL drops: (a) formamide on PMMA, (b) water on PMMA, (c) formamide on Teflon, and (d) water on Teflon.

In the 20–40 μL range, the top-view method became unreliable, with 0–1 out of 10 measurements falling within ±2° of the tangent method (Figure 9), regardless of contact angle. For example, water on Teflon showed a top-view average of 106.3°, while side-view methods exceeded 120°, with significant statistical differences (*p* < 0.01; Figure S8). These discrepancies stem from greater gravitational flattening, making the spherical cap assumption invalid. While ellipse fit and LB-ADSA remained consistent, top-view and circle fit methods showed frequent outliers, especially for θ_*s*_ > 90°. These findings confirm that the top-view method is volume-dependent and only reliable for small, spherical-cap-dominant drops.

**Figure 9:**
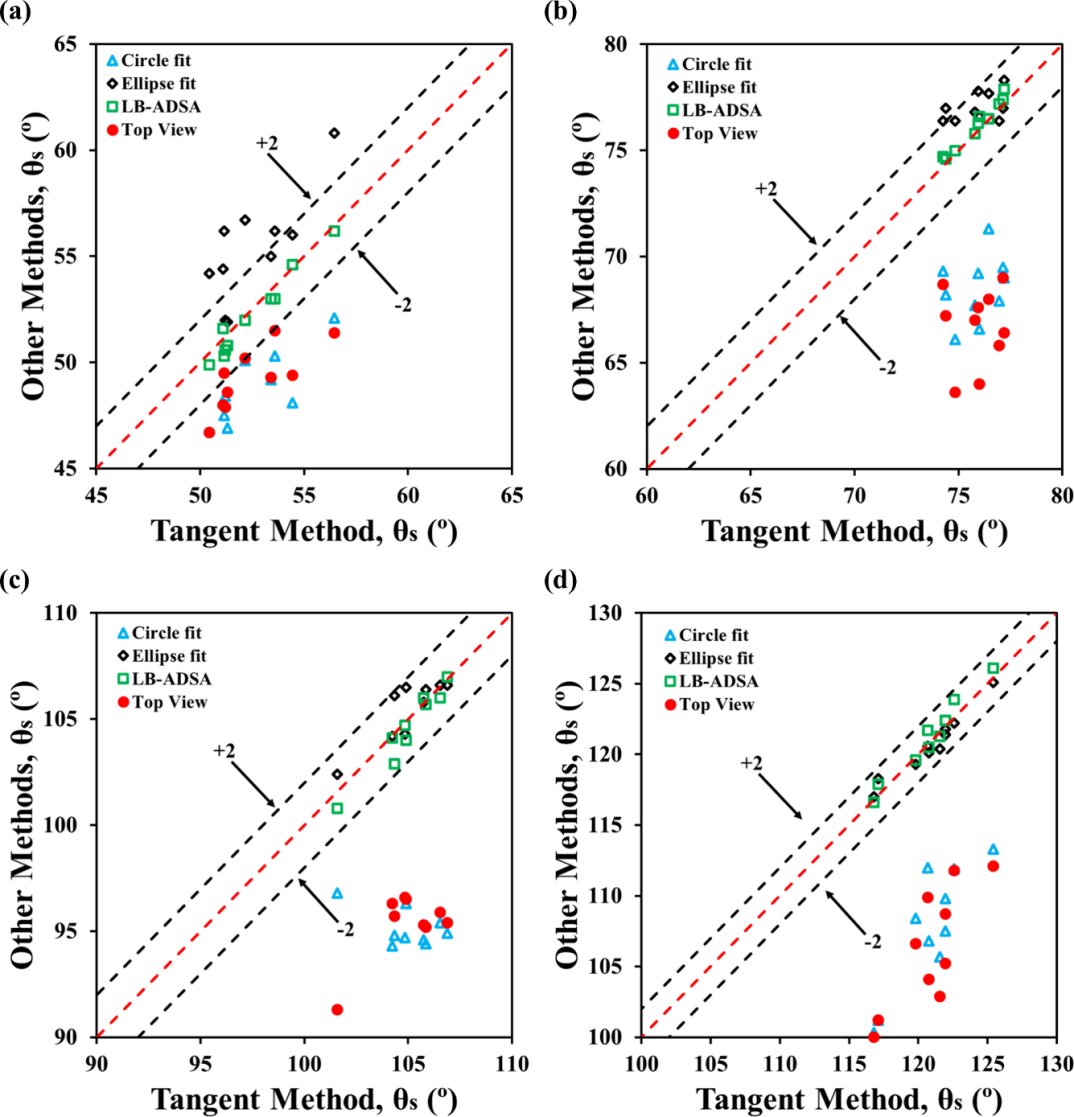
Performance of the top-view method against conventional side-view methods for 20–40 μL drops: (a) formamide on PMMA, (b) water on PMMA, (c) formamide on Teflon, and (d) water on Teflon.

### Geometric Shape Parameters

To quantify the influence of gravity on drop shape by comparing gravitational and surface tension forces, the Bond number (*B*_*o*_) is commonly used. In its expression (eq 3a), ρ and γ are, respectively, the density and surface tension of the liquid, *g* is the gravitational acceleration, *L* is the drop characteristic length (i.e., *R*_*o*_), and *l*_*cap*_ is the capillary length of the fluid defined as $\sqrt{\frac{\gamma }{\rho g}},$ which is 2.7 mm and 2.3 mm for water and formamide, respectively.

3a $$B_{o}=\frac{{\rho gL}^{2}}{\gamma }$$

or

3b $$B_{o}={\left(\frac{R_{o}}{l_{\mathrm{cap}}}\right)}^{2}\mathrm{when}L=R_{o}$$

*B*_*o*_ ≪ 1 indicates dominance of surface tension and a near-spherical cap shape. While Bo effectively characterizes side-view deformation, its applicability to top-view profiles is unclear. To address this top-view profile deformation, we introduce the Drop Projection Index (DPI), a modified *Bo* (eqs 4 or 5) by relating the gravitational force (mg) of the drop to the vertical component of surface force (2p*a* gsin*q*_*s*_) exerted by the liquid drop resting on a solid surface. DPI incorporates contact angle and uses top-view radius (*a* for θ_*s*_ < 90°, *R* for θ_*s*_ > 90°) as the characteristic length. It enables assessment of gravitational effects on top-view geometry and contact angles, though it does not account for contact line pinning, an area that needs future exploration.

4 $$\mathrm{DPI}=\frac{mg}{2\pi a\left(\gamma \sin\theta_{s}\right)}=\frac{\rho ga^{2}\left(2-3\cos\theta_{s}+\cos^{3}\theta_{s}\right)}{6\gamma \sin^{4}\theta_{s}},{\mathrm{for}\theta }_{s}<90^{◦}$$

5 $$\mathrm{DPI}=\frac{mg}{2\pi a\left(\gamma \sin\theta_{s}\right)}=\frac{\rho gR^{2}\left(2-3\cos\theta_{s}+\cos^{3}\theta_{s}\right)}{6\gamma \sin^{2}\theta_{s}},{\mathrm{for}\theta }_{s}>90^{◦}$$

Figure 10 summarizes the Drop Projection Index (DPI) thresholds for all fluid–sample combinations. The DPI (expected) is predicted from assumed perfect spherical caps of a given drop volume, while experimental DPI values were calculated using eqs 4 or 5, respectively, based on measured *a* or *R* values and the average contact angles for the 1–5 μL range. Deviations of experimental and expected DPI values appeared for large drops, but the thresholds varied by system: for θ_*s*_ < 90°, DPI was ∼0.15 for both formamide and water on PMMA, while for θ_*s*_ > 90°, DPI ranged from ∼0.2 (formamide on Teflon) to ∼0.3 (water on Teflon). These results support DPI as a more suitable geometric descriptor than the Bond number (*B*_*o*_) for top-view analysis, with *B*_*o*_ capturing side-view flattening and DPI reflecting top-view profile distortion. However, since DPI requires knowing the contact angle, we recommended performing DPI estimations as an additional step to verify if the measured contact angle is free of the influence of gravity or drop pinning.

**Figure 10:**
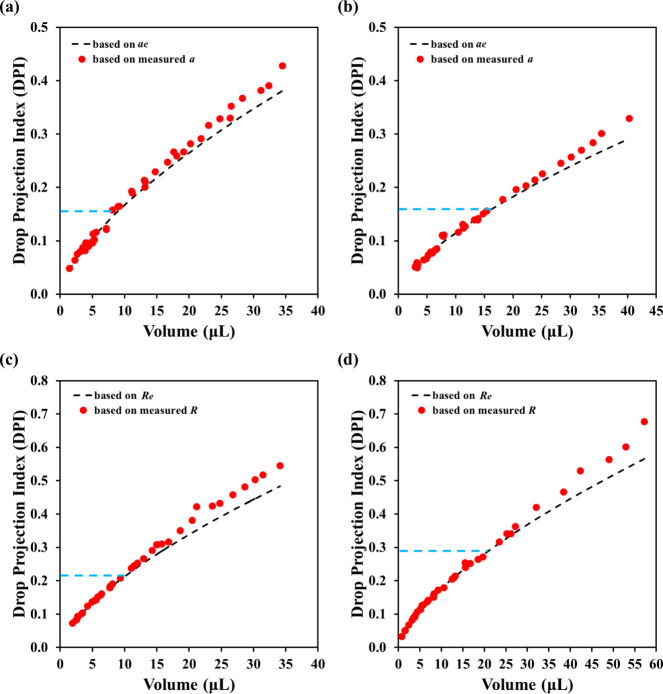
Variation of modified bond numbers and Drop Projection Index (DPI) with drop volume for (a) formamide on PMMA, (b) water on PMMA, (c) formamide on Teflon, and (d) water on Teflon.

While this study has been focused on how to choose suitable drop size when adopting the top-view method, besides avoiding drops greater than the threshold values, several additional limitations or practical considerations should be noted. Because small uncertainties in measured radii (a or R) can lead to large errors, especially for θ_*s*_ > 90°, care must be taken to accurately track the drop boundary and reliably determine the radii. The method also requires a reliable mass or volume measurement device to ensure accurate drop volume determination. It is not suited for probe liquids with low boiling points or high evaporation rates, as rapid evaporation can easily alter drop volume and in some cases, the drop shape during data acquisition. In addition, the method becomes challenging for high density probe liquids, which have small capillary lengths; in such cases, very small drops (typically 2–3 μL) must be used to minimize gravitational effects. During drop deposition, a low-adhesion needle or pipet tip is essential to ensure clean detachment of the drop and to avoid liquid retention on the dispensing tip. The method further requires a camera system capable of capturing drop geometry accurately without optical distortion, along with image-analysis software that can reliably resolve the drop boundary. Finally, the current implementation is restricted to static or quasi-static contact angle measurements and is not applicable to dynamic contact angle characterization.

## Conclusion

This study evaluated the top-view method as a practical alternative to conventional side-view techniques for obtaining static contact angles, particularly when side imaging is impractical or limited. Using a dual-camera setup and precise drop volume determination, the top-view method based on the spherical cap assumption was able to obtain reasonable contact angles of formamide and water on PMMA and Teflon, especially for small drop volume (<10 mL). As volume increased beyond 10 μL, gravitational flattening and contact line pinning of the drop, especially for contact angles above 90°, rendering the method less reliable. The Drop Projection Index (DPI) was introduced as a more suitable geometric descriptor than the Bond number for assessing gravitational effects in top-view imaging. Primary limitations of the top-view method included sensitivity to radius estimation, symmetry assumptions, and reduced accuracy for larger or nonwetting drops.

## Supplementary Material



## Data Availability

Data used in this study will be made available upon request.

## Notes

(Figure S1) Radius-contact angle relationship showing sensitivity of top-view method to both radius and contact angle regime; (Figures S2–S4) additional representative top and side-view images of probe fluids on PMMA and Teflon surfaces; (Table S1) advancing contact angle measurements of fluids on surfaces; (Figures S5–S8) t test p-value heatmaps showing statistical validations; (Figure S9) illustration of some primary forces acting on a liquid drop resting on a solid surface (PDF)

## †

Department of Petroleum and Natural Gas Engineering, New Mexico Institute of Mining and Technology, 801 Leroy Place, Socorro, NM 87801, United States

## References

[1] Akbari R., Antonini C. (2021). Contact Angle Measurements: From Existing Methods to
an Open-Source Tool. Adv. Colloid Interface
Sci..

[2] Chae H.-R., Lee J., Lee C. H., Kim I. C., Park P. K. (2015). Graphene Oxide-Embedded
Thin-Film Composite Reverse Osmosis Membrane with High Flux, Anti-Biofouling,
and Chlorine Resistance. J. Membr. Sci..

[3] Li M., Liu M., Qi F., Lin F. R., Jen A. K.-Y. (2024). Self-Assembled
Monolayers for Interfacial Engineering in Solution-Processed Thin-Film
Electronic Devices: Design, Fabrication, and Applications. Chem. Rev..

[4] Shen Y., Hu L., Chai W., Miao J., Ye H., Xu J., Su R., Fu X. (2025). A Mini Review on Contamination Control in Ultrapure
Liquids for Semiconductor Manufacturing - From the Perspective of
Liquid-Solid Interfaces. Flow Meas. Instrum..

[5] Andrews G. P., Laverty T. P., Jones D. S. (2009). Mucoadhesive
Polymeric Platforms
for Controlled Drug Delivery. Eur. J. Pharm.
Biopharm..

[6] Farkas D., Madarász L., Nagy Z. K., Antal I., Kállai-Szabó N. (2021). Image Analysis:
A Versatile Tool in the Manufacturing and Quality Control of Pharmaceutical
Dosage Forms. Pharmaceutics.

[7] Rossi, D. ; Pittia, P. ; Realdon, N. Contact Angle Measurements and Applications in Pharmaceuticals and Foods: A Critical Review. In Progress in Adhesion and Adhesives; Mittal, K. L. , Ed.; John Wiley & Sons Inc.: Hoboken, NJ, 2019; Vol. 4; pp 193–223.

[8] Croll S. G. (2020). Surface
Roughness Profile and Its Effect on Coating Adhesion and Corrosion
Protection: A Review. Prog. Org. Coat..

[9] Hang J., Yan X., Li J. (2024). A Review on
the Effect of Wood Surface Modification
on Paint Film Adhesion Properties. Coatings.

[10] Peng X., Zhang Z. (2019). Improvement of Paint
Adhesion of Environmentally Friendly Paint Film
on Wood Surface by Plasma Treatment. Prog. Org.
Coat..

[11] Ghosh J., Rupanty N. S., Noor T., Asif T. R., Islam T., Reukov V. (2025). Functional Coatings for Textiles: Advancements in Flame
Resistance, Antimicrobial Defense, and Self-Cleaning Performance. RSC Adv..

[12] He B., Hou X., Liu Y., Hu J., Song L., Tong Z., Zhan X., Ren Y., Liu Q., Zhang Q. (2023). Design of
Fluorine-Free Waterborne Fabric Coating with Robust Hydrophobicity,
Water-Resistant and Breathability. Sep. Purif.
Technol..

[13] Li S., Huang J., Chen Z., Chen G., Lai Y. (2017). A Review on
Special Wettability Textiles: Theoretical Models, Fabrication Technologies
and Multifunctional Applications. J. Mater.
Chem. A.

[14] Beketov G. V., Shynkarenko O. V. (2022). Surface Wetting and Contact Angle: Basics and Characterisation. Chem. Phys. Technol. Surf..

[15] Lamour G., Hamraoui A., Buvailo A., Xing Y., Keuleyan S., Prakash V., Eftekhari-Bafrooei A., Borguet E. (2010). Contact Angle Measurements
Using a Simplified Experimental Setup. J. Chem.
Educ..

[16] Xue C., Lott J. T., Kolachalama V. B. (2019). Estimation of Size and Contact Angle
of Evaporating Sessile Liquid Drops Using Texture Analysis. Langmuir.

[17] Mack G. L. (1936). The Determination
of Contact Angles from Measurements of the Dimensions of Small Bubbles
and Drops. I. The Spheroidal Segment Method for Acute Angles. J. Phys. Chem..

[18] Neumann, A. W. ; Good, R. J. Techniques of Measuring Contact Angles. In Surface and Colloid Science: Experimental Methods; Good, R. J. , Stromberg, R. R. , Eds.; Springer: Boston, MA, 1979; Vol. 11, pp 31–91.

[19] Mack G. L., Lee D. A. (1936). The Determination of Contact Angles
from Measurements
of the Dimensions of Small Bubbles and Drops. II. The Sessile Drop
Method for Obtuse Angles. J. Phys. Chem..

[20] Lubarda V. A., Talke K. A. (2011). Analysis of the Equilibrium Droplet Shape Based on
an Ellipsoidal Droplet Model. Langmuir.

[21] Bateni A., Susnar S. S., Amirfazli A., Neumann A. W. (2003). A High-Accuracy
Polynomial Fitting Approach to Determine Contact Angles. Colloids Surf. A Physicochem. Eng. Asp..

[22] Stalder A. F., Kulik G., Sage D., Barbieri L., Hoffmann P. (2006). A Snake-Based
Approach to Accurate Determination of Both Contact Points and Contact
Angles. Colloids Surf. A Physicochem. Eng. Asp..

[23] Skinner F. K., Rotenberg Y., Neumann A. W. (1989). Contact Angle Measurements from the
Contact Diameter of Sessile Drops by Means of a Modified Axisymmetric
Drop Shape Analysis. J. Colloid Interface Sci..

[24] Moy E., Cheng P., Policova Z., Treppo S., Kwok D., Mack D. P., Sherman P. M., Neuman A. W. (1991). Measurement of Contact
Angles from the Maximum Diameter of Non-wetting Drops by Means of
a Modified Axisymmetric Drop Shape Analysis. Colloid surf..

[25] Stalder A. F., Melchior T., Müller M., Sage D., Blu T., Unser M. (2010). Low-Bond Axisymmetric
Drop Shape Analysis for Surface Tension and
Contact Angle Measurements of Sessile Drops. Colloids Surf. A Physicochem. Eng. Asp..

[26] Hoorfar M., Neumann A. W. (2006). Recent Progress
in Axisymmetric Drop Shape Analysis
(ADSA). Adv. Colloid Interface Sci..

[27] Bikerman J. (1941). Method of
Measuring Contact Angles. Ind. Eng. Chem. Anal.
Ed..

[28] Birdi K. S., Vu D. T., Winter A. (1989). A Study of the Evaporation Rates
of Small Water Drops Placed on a Solid Surface. J. Phys. Chem..

[29] Picknett R. G., Bexon R. (1977). The Evaporation of
Sessile or Pendant Drops in Still Air. J. Colloid
Interface Sci..

[30] Rowan S. M., Newton M. I., McHale G. (1995). Evaporation of Microdroplets
and
the Wetting of Solid Surfaces. J. Phys. Chem..

[31] Stauber J. M., Wilson S. K., Duffy B. R., Sefiane K. (2015). Evaporation of Droplets
on Strongly Hydrophobic Substrates. Langmuir.

[32] Dutra G., Martelli C., Canning J. (2015). Simple Top Down Imaging Measurement
of Contact Angle for Practical Assessment of Hydrophilic Surfaces. Proc. SPIE.

[33] Zhang N., Chao D. F. (2002). A New Laser Shadowgraphy
Method for Measurements of
Dynamic Contact Angle and Simultaneous Flow Visualization in a Sessile
Drop. Opt. Laser Technol..

[34] Janeczko C., Martelli C., Canning J., Dutra G. (2019). Assessment of Orchid
Surfaces Using Top-Down Contact Angle Mapping. IEEE Access.

[35] McHale G., Erbil H. Y., Newton M. I., Natterer S. (2001). Analysis of Shape Distortions
in Sessile Drops. Langmuir.

[36] Rodrıguez-Valverde M. A., Cabrerizo-Vılchez M. A., Rosales-López P., Páez-Dueñas A., Hidalgo-Álvarez R. (2002). Contact Angle
Measurements on Two (Wood and Stone) Non-Ideal Surfaces. Colloids Surf. A Physicochem. Eng. Asp..

[37] Schuster J. M., Schvezov C. E., Rosenberger M. R. (2015). Influence
of Experimental Variables
on the Measure of Contact Angle in Metals Using the Sessile Drop Method. Procedia Mater. Sci..

[38] Jańczuk B., Wójcik W., Zdziennicka A. (1993). Determination
of the Components of
the Surface Tension of Some Liquids from Interfacial Liquid-liquid
Tension Measurements. J. colloid interface sci..

[39] Zdziennicka A., Szymczyk K., Krawczyk J., Jańczuk B. (2017). Some Remarks
on the Solid Surface Tension Determination from Contact Angle Measurements. Appl. Surf. Sci..

[40] Schneider C. A., Rasband W. S., Eliceiri K. W. (2012). NIH Image to ImageJ:
25 Years of
Image Analysis. Nat. methods.

[41] Rios P. F., Dodiuk H., Kenig S., McCarthy S., Dotan A. (2007). The Effect
of Polymer Surface on the Wetting and Adhesion of Liquid Systems. J. Adhes. Sci. Technol..

[42] Karhan M. (2021). Experimental
Investigation of Wettability and Evaporation for the Surface of PMMA
Dielectric Material Used in High-Voltage Applications and Outdoor
Electrical Applications. Appl. Phys. A: Mater.
Sci. Process..

[43] Extrand C. W., Moon S. I. (2010). When Sessile Drops Are No Longer
Small: Transitions
from Spherical to Fully Flattened. Langmuir.

[44] Tonini S., Cossali G. E. (2024). Modeling the Effect
of Shape Deformation Induced by
Gravity on the Evaporation of Pendant and Sessile Drops. Phys. Fluids.

[45] Zhao X., Xie F., Fan J., Liu D., Huang J., Chen S. (2018). Evaluation
on Dorsey Method in Surface Tension Measurement of Solder Liquids
Containing Surfactants. Int. J. Thermophys..

[46] Dorsey N. E. (1928). A New Equation
for the Determination of Surface Tension from the Form of a Sessile
Drop or Bubble. J. Wash. Acad. Sci..

[47] Eral H. B., ’t Mannetje D. J. C. M., Oh J. M. (2013). Contact Angle Hysteresis:
A Review of Fundamentals and Applications. Colloid
Polym. Sci..

[48] Lam C. N. C., Kim N., Hui D., Kwok D. Y., Hair M. L., Neumann A. W. (2001). The Effect of Liquid Properties to
Contact Angle Hysteresis. Colloids Surf. A Physicochem.
Eng. Asp..

