# Supplementary material for: Top-View Method as a Robust Alternative for Contact Angle Measurement
Source: Langmuir. 2026 Mar 4;42(10):7060–72. doi: 10.1021/acs.langmuir.5c04532 (PMC13001083; doi:10.1021/acs.langmuir.5c04532)
Supplement: Supplementary file 1 [file la5c04532_si_001.pdf]

Supporting Information

Top-View Method as a Robust Alternative for Contact Angle Measurement

Emmanuel Agyei and Bi-min Zhang Newby

Department of Chemical, Biomolecular, and Corrosion Engineering, The University of Akron, Akron, OH, 44325-3906, United States

1.0 Sensitivity of the top-view method

Figure S1 illustrates the relationship between static contact angle and top-view radius measurements derived analytically from the spherical cap model. For drops with contact angles less than 90°, the measured radius corresponds to the base radius, while for contact angles greater than 90°, the radius refers to the maximum horizontal projection of the drop, often termed the "radius of the greater circle". These relationships were constructed for theoretical drop volumes of 2 µL, 7 µL, 15 µL, 30 µL, and 50 µL, using exact analytical solutions of the spherical cap volume formula as a function of contact angle and radius. As shown in the plot, for all volume categories, contact angle estimation is more sensitive to changes in the top-view radius when the contact angle is greater than 90° (represented by dashed lines), compared to when it is less than 90° (solid lines). This heightened sensitivity for angles above 90° is evidenced by the steeper slope of the dashed curves: small changes in the measured radius result in large variations in the estimated contact

angle. Consequently, the top-view technique must achieve precise radius measurements to avoid significant estimation errors in this regime. For contact angles less than 90°, sensitivity to radius measurements gradually increases as the contact angle approaches 90°, as indicated by the tightening curvature of the solid lines. This suggests that for low to moderate contact angles, such as 45° to 70°, minor deviations in measured base radius produce relatively small errors in contact angle estimation. However, the method becomes progressively more sensitive (small inaccuracies in radius measurements can cause very significant errors in contact angle estimation), closer to the 90° threshold. In contrast, for contact angles exceeding 150°, the top-view method becomes practically unusable. At this stage, the analytical relationship between radius and contact angle plateaus. That is, the radius values required to distinguish between, for example, 150°, 160°, and 170°, become so similar (identical up to four decimal places) that it becomes impossible to distinguish the actual contact angle based on top-view radius alone. This degeneracy implies that even extremely small experimental uncertainties in radius measurement would yield unreliable or meaningless angle estimates. These initial findings served as a guide for further objectives in the study.

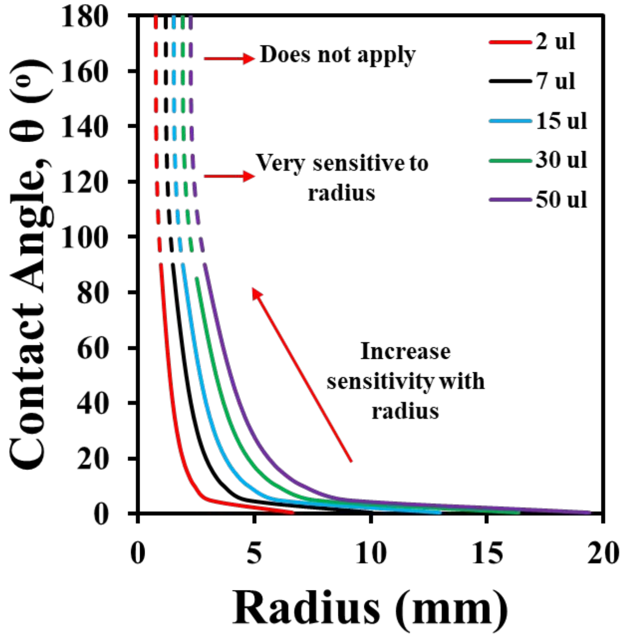

Figure S1. Sensitivity of the spherical cap model to top-view radius measurements for various drop volumes. The curves represent the analytical relationship between contact angle (θ) and measured radius (base radius a for θ < 90° shown with solid lines, and maximum radius R for θ > 90° shown with dashed lines) for drop volumes of 2 µL, 7 µL, 15 µL, 30 µL, and 50 µL. Results demonstrate that contact angle estimation becomes increasingly sensitive to radius measurements as θ increases, particularly for θ > 90°, where small errors in radius (R) can lead to significant inaccuracies in angle prediction.

2.0 Additional Representative Images

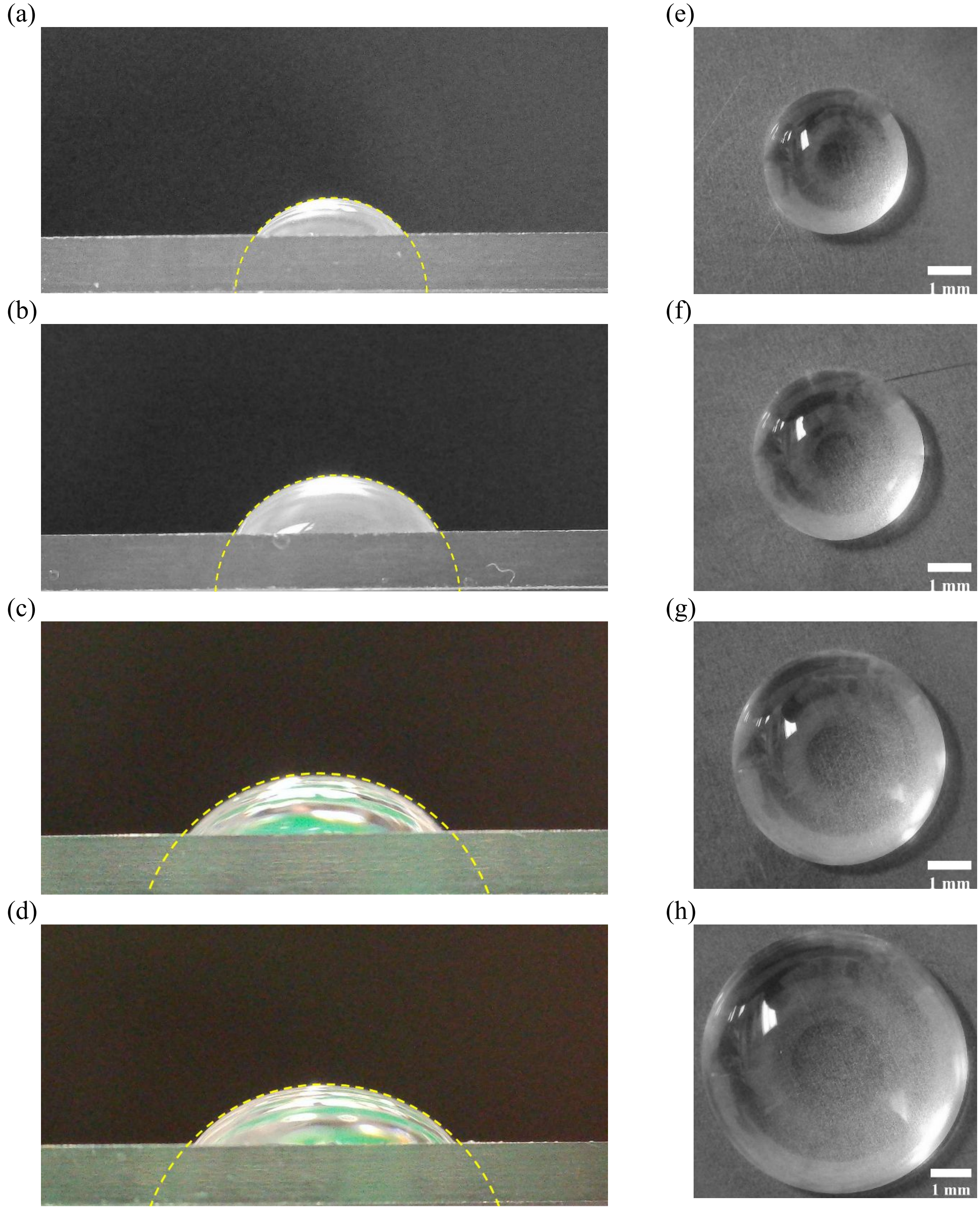

Figure S2. Typical side-view and corresponding top-view images of 2.7μL (a & e), 7.1μL (b & f), 16.7μL (c & g), and 32.1μL (d & h) formamide drops on a PMMA surface.

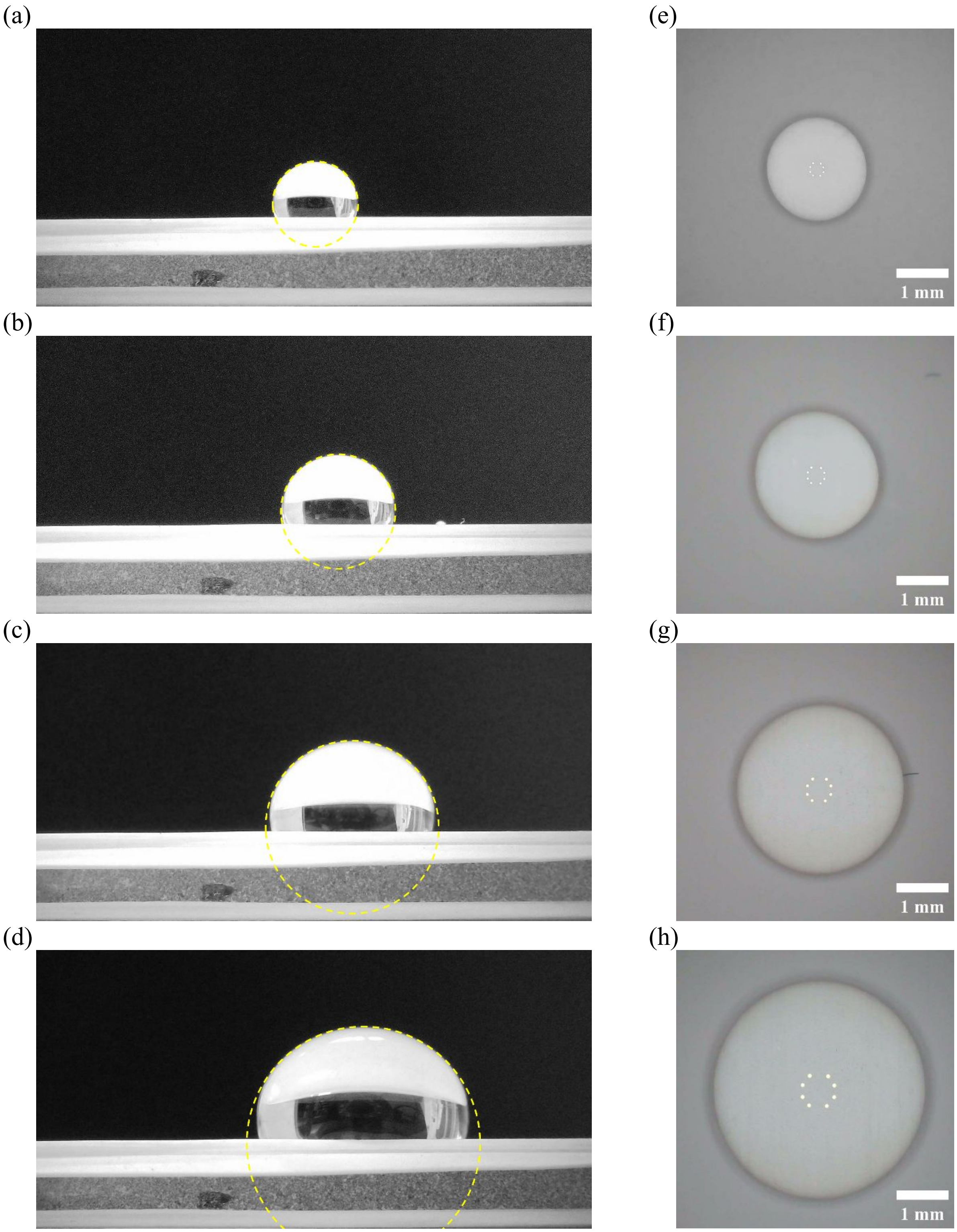

Figure S3. Typical side-view and corresponding top-view images of 2.9μL (a & e), 7.7μL (b & f), 15μL (c & g), and 30.3μL (d & h) formamide drops on a Teflon surface.

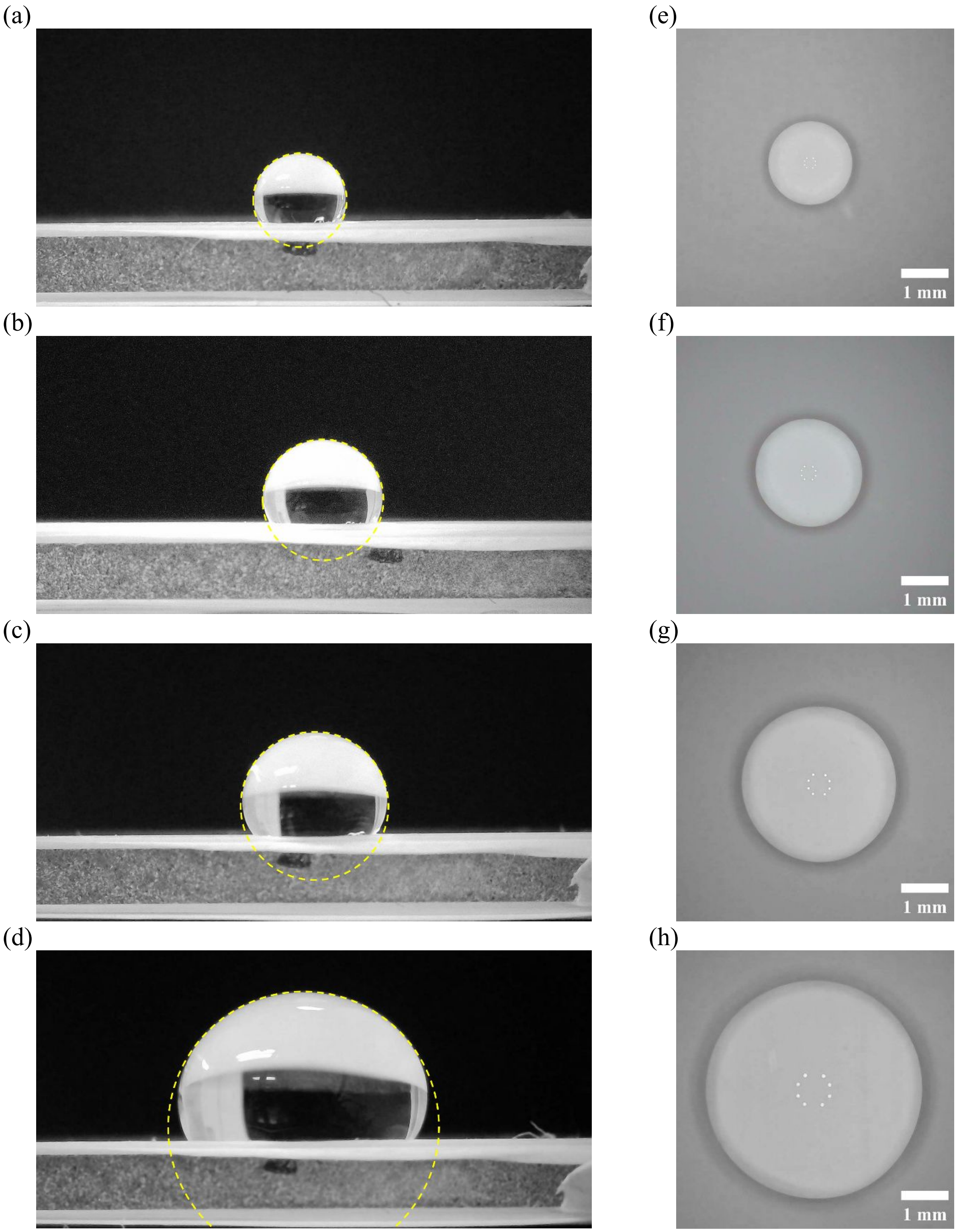

Figure S4. Typical side and corresponding top view images of 2.4μL (a & e), 5.1μL (b & f), 12.6μL (c & g), and 42.3μL (d & h) water drops on a Teflon surface.

Figures S2, **S3**, and **S4** show additional representative images of side-view and corresponding top-view images of formamide on PMMA, formamide on Teflon, and water on Teflon, respectively. All drops in the different volume groups exhibit similar behavior compared to what was thoroughly discussed in the main text (water on PMMA).

Table S1. Side view static and advancing contact angle measurements of all fluid-substrate systems using a standard goniometer.

**Fluid (Substrate)**	θ_s (°)	θ_{adv} (°)
Formamide (PMMA)	49.9 ± 1.8	60.4 ± 1.2
Water (PMMA)	66.9 ± 1.7	75.1 ± 1
Formamide (Teflon)	102.5 ± 1.1	116.8± 0.7
Water (Teflon)	109.8 ± 2.2	123.0 ± 2.1

Table S1 summarizes the measured advancing and static contact angles of formamide and water on all samples, obtained using a standard goniometer setup to assess how these liquids interact with the sample surfaces. The advancing contact angles were measured from side-view imaging and measured separately to verify if the values correlate with the top-view contact angle measurements.

3.0 Statistical Analysis

Figures S5 to S8 show statistical heatmaps (results) used to assess the comparative performance of the top-view method against conventional side-view methods. As stated in the main text, the tangent method was adopted as the primary reference for this study. Hence, all methods were compared statistically against the top-view method and the tangent method. Information derived from the statistical analysis of the measured data is thoroughly presented in the main text.

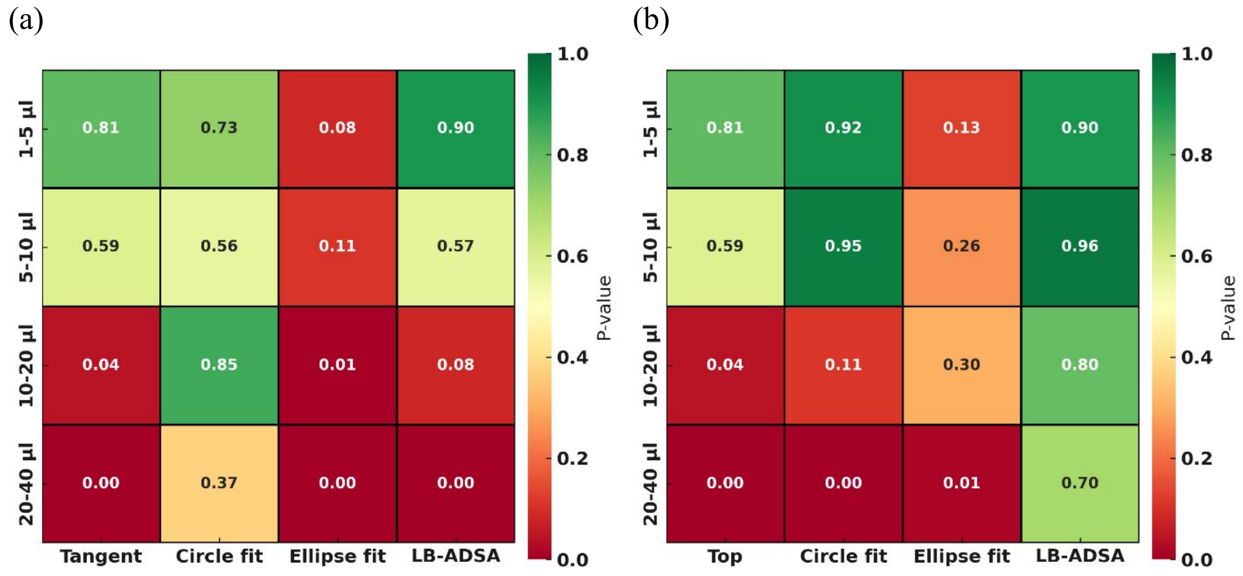

Figure S5: Two-sample t-test p-value heatmaps for formamide on PMMA. (a) comparing all methods with the top view method and (b) all methods with the tangent method across four volume groups. Green indicates no significant difference ($p \approx 1$), while red indicates a high significance difference ($p \approx 0$). The top view shows good agreement with side-view methods at small volumes but increasingly diverges at larger volumes.

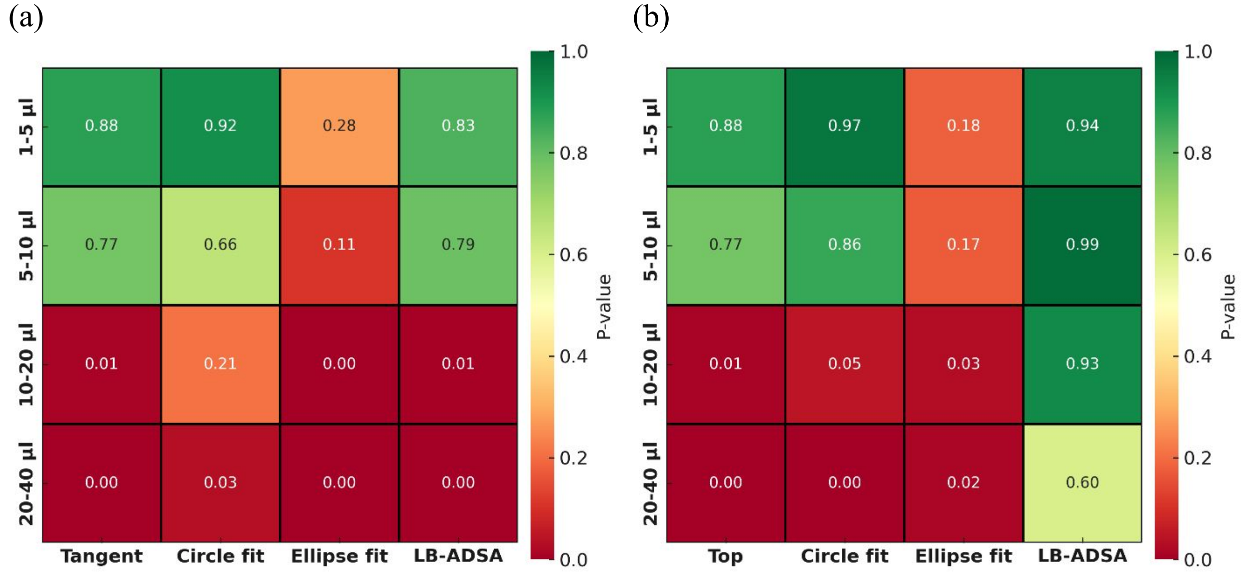

Figure S6: Two-sample t-test p-value heatmaps for water on PMMA. (a) Comparisons between the top-view method and other techniques; (b) Comparisons between the tangent method and other techniques. Warmer colors indicate lower p-values and higher statistical significance in observed differences.

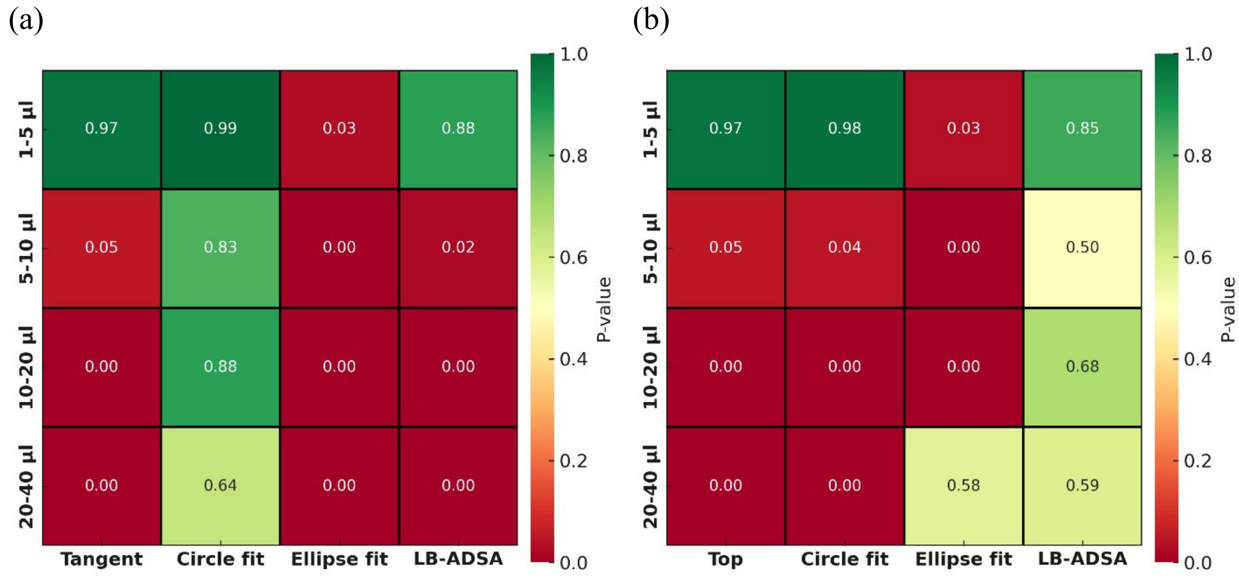

Figure S7: Two-sample t-test p-value heatmaps for formamide on Teflon. (a) Comparisons between the top-view method and other techniques; (b) Comparisons between the tangent method and other techniques. Warmer colors indicate lower p-values and higher statistical significance in observed differences.

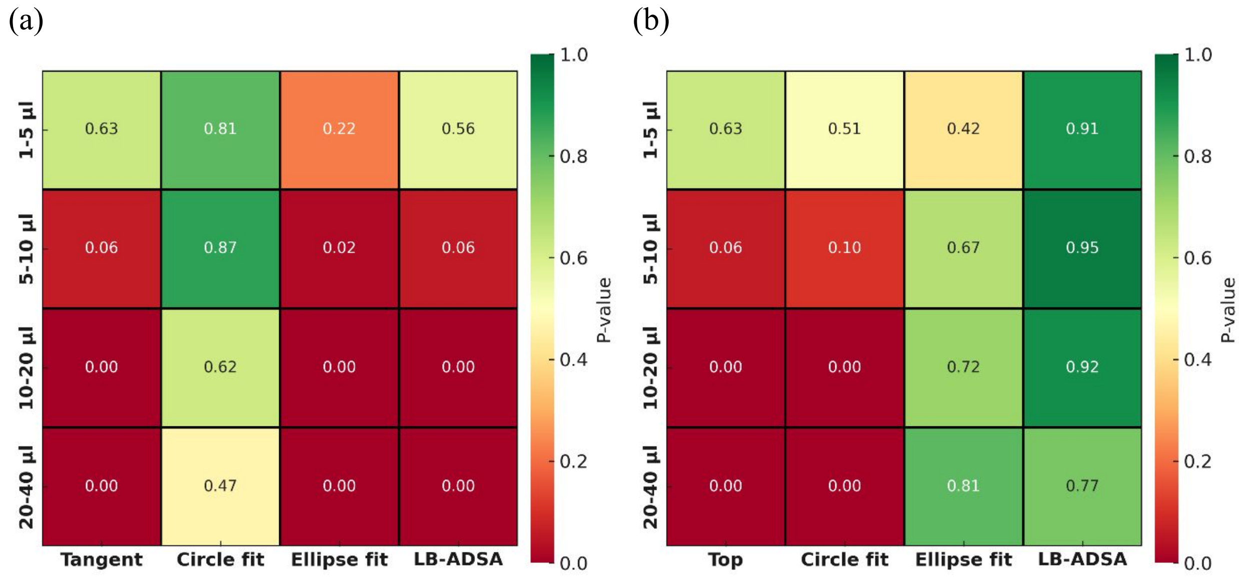

Figure S8: Two-sample t-test p-value heatmaps for water on Teflon. (a) Comparisons between the top-view method and other techniques; (b) Comparisons between the tangent method and other techniques. Warmer colors indicate lower p-values and higher statistical significance in observed differences.

4.0 Derivation of Drop Projection Index (DPI)

For a liquid drop resting on a solid surface under equilibrium conditions, two primary vertical forces that are relevant to this study are: (1) the vertical component of the surface tension acting along the three-phase contact line, given by $2\pi a\ \gamma\sin\theta_s$,and (2) the gravitational force, *mg*, as illustrated in the schematic below. In this study, we define a dimensionless parameter, DPI, as the ratio of these two forces:

$$\text{DPI} = \frac{mg}{2\pi a\ \gamma sin\theta_s} \quad \text{(S1a)}$$

This parameter is used to quantify the influence of gravity on drop flattening and to to identify the conditions under which the spherical-cap approximation remains valid.

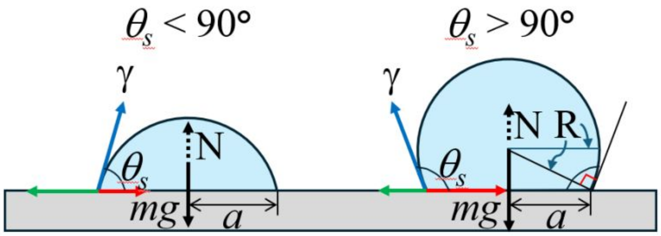

Figure S9. The sketch illustrates some primary forces acting on a liquid drop resting on a solid surface. The details shown on the right demonstrate how the base radius, *a*, and the maximum horizontal radius, R, are related through basic trigonometric relations when $\theta_s > 90°$. Specially, $a=R\cos(\theta_s - 90°)=R\cos(90° - \theta_s)=R\sin\theta_s$.

Using the geometrical relations of spherical-cap drops (Figure 1) together with trigonometric relations, DPI can be expressed in terms of the wetted radius *a* and θ_s for $\theta_s < 90°$ and in terms of R and θ_s for $\theta_s > 90°$. The detailed deriviations of the corresponding DPI expression are presented below.

$$\text{DPI} = \frac{mg}{2\pi a(\gamma sin\theta_s)} = \frac{\rho gV}{2\pi a(\gamma sin\theta_s)} \quad \text{(S1b)}$$

For $\theta_s < 90°$

$$V = \frac{\pi a^3(2 - 3cos\theta_s + cos^3\theta_s)}{3sin^3\theta_s} \quad (1)$$

$$\text{DPI} = \frac{\rho g a^2(2-3\,cos\,\theta_s+cos^3\theta_s)}{6\gamma sin^4\theta_s} \quad \text{(S1c)}$$

For $\theta_s > 90^o$

$$V = \frac{\pi R^3}{3}(2 - 3\cos\theta_s + cos^3\theta_s) \quad (2)$$

$$\text{DPI} = \frac{\rho g V}{2\pi a(\gamma sin\theta_s)} = \frac{\rho g V}{2\pi(R sin\theta_s)(\gamma sin\theta_s)} = \frac{\rho g R^2(2 - 3\,cos\,\theta_s + cos^3\theta_s)}{6\gamma sin^2\theta_s} \quad \text{(S1d)}$$

where $a = R\ cos(\theta_s\ \text{-}90°)\text{=}R\ cos(90°\ \text{-}\ \theta_s)\text{=}R\ sin\ \theta_s$
